# A Comprehensive Framework for Saturation Theorem Proving

**DOI:** 10.1007/s10817-022-09621-7

**Published:** 2022-06-07

**Authors:** Uwe Waldmann, Sophie Tourret, Simon Robillard, Jasmin Blanchette

**Affiliations:** 1 Max-Planck-Institut für Informatik, Saarland Informatics Campus, Saarbrücken, Germany; 2 Université de Lorraine, CNRS, Inria, LORIA, Nancy, France; 3 LIRMM, Université de Montpellier, CNRS, Montpellier, France; 4 Vrije Universiteit Amsterdam, Amsterdam, the Netherlands

**Keywords:** Automated theorem proving, Saturation, Resolution calculus, Superposition calculus, Redundancy, Prover architectures

## Abstract

A crucial operation of saturation theorem provers is deletion of subsumed formulas. Designers of proof calculi, however, usually discuss this only informally, and the rare formal expositions tend to be clumsy. This is because the equivalence of dynamic and static refutational completeness holds only for derivations where all deleted formulas are redundant, but the standard notion of redundancy is too weak: A clause *C* does not make an instance $C\sigma$ redundant. We present a framework for formal refutational completeness proofs of abstract provers that implement saturation calculi, such as ordered resolution and superposition. The framework modularly extends redundancy criteria derived via a familiar ground-to-nonground lifting. It allows us to extend redundancy criteria so that they cover subsumption, and also to model entire prover architectures so that the static refutational completeness of a calculus immediately implies the dynamic refutational completeness of a prover implementing the calculus within, for instance, an Otter or DISCOUNT loop. Our framework is mechanized in Isabelle/HOL.

## Introduction

Saturation is one of the most successful approaches to automatic theorem proving. Provers based on resolution, superposition, and related proof calculi all implement some saturation procedure that systematically derives conclusions of inferences up to a redundancy criterion. More specifically, a saturation prover starts with a problem to refute, typically given as a set of clauses, and draws inferences from the clauses, adding the conclusions to the set. The prover may remove redundant clauses at any point. The refutation attempt ends as soon as the empty clause $\bot$, denoting a contradiction, has been derived. The proof calculus specifies which inferences need to be performed and which clauses are redundant.

In their *Handbook of Automated Reasoning* chapter [6, Sect. 4], Bachmair and Ganzinger remark that “unfortunately, comparatively little effort has been devoted to a formal analysis of redundancy and other fundamental concepts of theorem proving strategies, while more emphasis has been placed on investigating the refutational completeness of a variety of modifications of inference rules, such as resolution.” As a remedy, they present an abstract framework for saturation up to redundancy. Briefly, theorem proving derivations take the form $N_0 \rhd N_1 \rhd \cdots$, where $N_0$ is the initial clause set and each step either adds inferred clauses or deletes redundant clauses. Given a suitable notion of fairness, the limit $N_\infty$ of a fair derivation is saturated up to redundancy. If the calculus is refutationally complete and $N_\infty$ does not contain $\bot$, then $N_0$ has a model. We will refer to the calculus’ refutational completeness as *static* as opposed to a prover’s *dynamic* completeness.

Bachmair and Ganzinger also define a concrete prover, $\mathsf {RP}$, based on a first-order ordered resolution calculus and the given clause procedure. However, like all realistic resolution provers, $\mathsf {RP}$ implements subsumption deletion: It will delete a clause *C* if it is subsumed by another clause $C'$, meaning that $C = C'\sigma \vee D$ for some substitution $\sigma$ and some clause *D*. Yet the case where $D = \bot$ is not covered by the standard definition of redundancy, according to which a clause *C* is redundant w.r.t. a clause set *N* if all its ground instances $C\theta$ are entailed by *strictly* smaller ground instances of clauses belonging to *N*. Concretely, we would like ${\mathsf {P}}(x)$ to make ${\mathsf {P}}({\mathsf {a}})$ redundant, but this fails because the instance of ${\mathsf {P}}(x)$ that entails ${\mathsf {P}}({\mathsf {a}})$, namely ${\mathsf {P}}({\mathsf {a}})$ itself, is not strictly smaller than ${\mathsf {P}}({\mathsf {a}})$. As a result, $\mathsf {RP}$-derivations are *not* $\rhd$-derivations, and Bachmair and Ganzinger’s saturation framework is *not* applicable.

There are two ways to address this problem. In the *Handbook*, Bachmair and Ganzinger start from scratch and prove the dynamic refutational completeness of $\mathsf {RP}$ by relating nonground derivations to ground derivations. This proof, though, turns out to be rather nonmodular—it refers simultaneously to properties of the calculus, to properties of the prover, and to the fairness of the derivations. Extending it to other calculi or prover architectures would be costly. For this reason, most authors stop after proving static refutational completeness of their calculi.

An alternative approach is to extend the redundancy criterion so that subsumed clauses become redundant. As demonstrated by Bachmair and Ganzinger in 1990 [3], this is possible by redefining redundancy in terms of closures $(C,\theta )$ instead of ground instances $C\theta$. We show that this approach can be generalized and modularized: First, any redundancy criterion that is obtained by lifting a ground criterion can be extended to a redundancy criterion that supports subsumption without affecting static refutational completeness. Second, by applying this property to labeled formulas, it becomes possible to give generic completeness proofs for prover architectures in a straightforward way.

Most saturation provers implement a variant of the given clause procedure. We present an abstract version of the procedure that can be refined to obtain an Otter [28] or DISCOUNT [1] loop, and we prove it refutationally complete. We also present a generalization that decouples scheduling and computation of inferences, to support *orphan formula deletion* and *inference dovetailing*. A formula is an orphan when it has lost one of its parents to the redundancy criterion [24, 39]; removing such formulas reduces the search space. Dovetailing makes it possible to support infinitary inference rules, such as $\lambda$-superposition and its possibly infinite sequences of higher-order unifiers [14].

When users of the framework instantiate these prover architectures with a concrete saturation calculus, they obtain the dynamic refutational completeness of the combination from the properties of the prover architecture and the static refutational completeness proof for the calculus. The framework is applicable to a wide range of calculi, including ordered resolution [6], unfailing completion [2], standard superposition [5], constraint superposition [29], theory superposition [44], and hierarchic superposition [8], It is already used in several published and ongoing works on combinatory superposition [15], $\lambda$-free superposition [12], $\lambda$-superposition [13, 14], superposition with interpreted Booleans [33], AVATAR-style splitting [21], and superposition with SAT-inspired inprocessing [43].

When Schlichtkrull, Blanchette, Traytel, and Waldmann [36] mechanized Bachmair and Ganzinger’s chapter using the Isabelle/HOL proof assistant [32], they found quite a few mistakes, including one that compromised $\mathsf {RP}$’s dynamic refutational completeness [36, Sect. 7.1]. This motivated us to mechanize our framework as well.

This article is structured as follows:

- Section 2 introduces the basic notions of Bachmair–Ganzinger-style saturation theorem proving, which also form the basis of our framework. These include the notions of inferences, redundancy, and static and dynamic refutational completeness.
- Section 3 shows how to lift the refutational completeness of a ground calculus to the nonground level. This step adds support for subsumption and formula labels.
- Section 4 presents several prover architectures that are variants of the given clause procedure, showing how to cope with multiple formula sets.
- Section 5 briefly describes the Isabelle mechanization of the framework.

An earlier version of this work was presented at IJCAR 2020 [45]. This article extends the IJCAR paper with detailed proofs, more explanations and examples, and an index. The Isabelle mechanization is described in more detail in a CPP 2021 paper [41].

## Preliminaries

Our framework is parameterized by abstract notions of formulas, inferences, and redundancy criteria, defined below. We also introduce various auxiliary concepts, notably static and dynamic refutational completeness, and study variations found in the literature.

### Inferences and Redundancy

A set ${\mathbf {F}}$ of *formulas* is a nonempty set with a nonempty subset ${\mathbf {F}}_\bot \subseteq {\mathbf {F}}$. Elements of ${\mathbf {F}}_\bot$ represent *false*. Typically, ${\mathbf {F}}_\bot$ is a singleton—i.e., ${\mathbf {F}}_\bot = \{\bot \}$. The possibility to distinguish between several *false* elements will be useful when we model concrete prover architectures, where different elements of ${\mathbf {F}}_\bot$ represent different situations in which a contradiction has been derived.

A *consequence relation* ${\models }$ over ${\mathbf {F}}$ is a relation ${\models } \subseteq \mathcal {P}({\mathbf {F}}) \times \mathcal {P}({\mathbf {F}})$ with the following properties for all $N_1, N_2, N_3 \subseteq {\mathbf {F}}$:

1. $$\{\bot \} \models N_1$$ for every $$\bot \in {\mathbf {F}}_\bot $$;
2. $$N_2 \subseteq N_1$$ implies $$N_1 \models N_2$$;
3. if $N_1 \models \{C\}$ for every $C \in N_2$, then $N_1 \models N_2$;
4. if $N_1 \models N_2$ and $N_2 \models N_3$, then $N_1 \models N_3$.

It is easy to show that $N_1 \models N_2$ if and only if $N_1 \models \{C\}$ for every $C \in N_2$, and that $N \models \bigcup _{i \in I} N_i$ if and only if $N \models N_i$ for every $i \in I$. Moreover, all elements of ${\mathbf {F}}_\bot$ are logically equivalent: If $N \models \{\bot \}$ for some $\bot \in {\mathbf {F}}_\bot$, then $N \models \{\bot '\}$ for every $\bot ' \in {\mathbf {F}}_\bot$.

Consequence relations are used (1) when one discusses the soundness of a calculus (and hence, when we justify the addition of formulas) and (2) when one discusses the refutational completeness of a calculus (and hence, when we justify the deletion of redundant formulas). Perhaps unexpectedly, the consequence relations used for these purposes may be different ones. A typical example is theory superposition, where one may use entailment w.r.t. all theory axioms for (1), but only entailment w.r.t. a subset of the (instances of the) theory axioms for (2). Another example is constraint superposition, where one uses entailment w.r.t. the set of all ground instances for (1), but entailment w.r.t. a subset of those instances for (2). Typically, the consequence relation  used for (1) is the intended one, and some additional calculus-dependent argument is necessary to show that refutational completeness w.r.t. the consequence relation $\models$ used for (2) implies refutational completeness w.r.t. .

An $${\mathbf {F}}$$*-inference* $$\iota $$ is a tuple $$(C_n,\ldots ,C_0) \in {\mathbf {F}}^{n+1}$$, $$n \ge 0$$. The formulas $$C_n,\ldots , C_1$$ are called *premises* of $$\iota $$; $$C_0$$ is called the *conclusion* of $$\iota $$, denoted by $$ concl (\iota )$$. An $${\mathbf {F}}$$-*inference system* $$ Inf $$ is a set of $${\mathbf {F}}$$-inferences. If $$N \subseteq {\mathbf {F}}$$, we write $$ Inf (N)$$ for the set of all inferences in $$ Inf $$ whose premises are contained in *N*. We write $$ Inf (N, M) := Inf (N \cup M) \setminus Inf (N \setminus M)$$ for the set of all inferences in $$ Inf $$ such that one premise is in *M* (and possibly in *N* as well) and the other premises are contained in $$N \cup M$$.

One can find several slightly differing definitions for redundancy criteria, fairness, and saturation in the literature [6, 8, 44]. We discuss the differences in Sect. 2.3. Here we mostly follow Waldmann [44].

A *redundancy criterion* for an inference system $Inf$ and a consequence relation ${\models }$ is a pair $Red = ( Red _{\text {I}}, Red _{\text {F}})$, where $Red _{\text {I}}: \mathcal {P}({\mathbf {F}}) \rightarrow \mathcal {P}( Inf )$ and $Red _{\text {F}}: \mathcal {P}({\mathbf {F}}) \rightarrow \mathcal {P}({\mathbf {F}})$ are mappings from sets of formulas to sets of inferences and from sets of formulas to sets of formulas that satisfy the following conditions for all sets of formulas *N* and $N'$:

1. if $N \models \{\bot \}$ for some $\bot \in {\mathbf {F}}_\bot$, then $N \setminus Red _{\text {F}}(N) \models \{\bot \}$;
2. if $N \subseteq N'$, then $Red _{\text {F}}(N) \subseteq Red _{\text {F}}(N')$ and $Red _{\text {I}}(N) \subseteq Red _{\text {I}}(N')$;
3. if $N' \subseteq Red _{\text {F}}(N)$, then $Red _{\text {F}}(N) \subseteq Red _{\text {F}}(N \setminus N')$ and $Red _{\text {I}}(N) \subseteq Red _{\text {I}}(N \setminus N')$;
4. if $\iota \in Inf$ and $concl (\iota ) \in N$, then $\iota \in Red _{\text {I}}(N)$.

Inferences in $Red _{\text {I}}(N)$ and formulas in $Red _{\text {F}}(N)$ are called *redundant* w.r.t. *N*. In a prover, $Red _{\text {I}}$ indicates which inferences need not be performed (e.g., because they have already been performed), whereas $Red _{\text {F}}$ justifies the deletion and simplification of formulas. Intuitively, (R1) states that deleting redundant formulas preserves inconsistency. (R2) and (R3) state that formulas or inferences that are redundant w.r.t. a set *N* remain redundant if arbitrary formulas are added to *N* or redundant formulas are deleted from *N*. (R4) ensures that computing an inference makes it redundant. Note that $C \in Red _{\text {F}}(\{C\})$ generally does not hold.

The connection between redundant inferences and redundant formulas is given by the second part of (R3). Together with (R4) it implies that inferences with a redundant conclusion are themselves redundant:

#### Lemma 1

If $\iota \in Inf$ and $concl (\iota ) \in Red _{\text {F}}(N)$, then $\iota \in Red _{\text {I}}(N)$.

#### Proof

Let $\iota \in Inf$ and $concl (\iota ) \in Red _{\text {F}}(N)$. Then $\iota \in Red _{\text {I}}( Red _{\text {F}}(N)) \subseteq Red _{\text {I}}(N \cup Red _{\text {F}}(N))$. Since $Red _{\text {F}}(N) \setminus N \subseteq Red _{\text {F}}(N) \subseteq Red _{\text {F}}(N \cup Red _{\text {F}}(N))$, we obtain $\iota \in Red _{\text {I}}(N \cup Red _{\text {F}}(N)) \subseteq Red _{\text {I}}((N \cup Red _{\text {F}}(N)) \setminus ( Red _{\text {F}}(N) \setminus N)) = Red _{\text {I}}(N)$. $\square$

Redundant inferences will play a central role in the definition of saturation and refutational completeness in Sect. 2.2.

#### Example 2

The *trivial redundancy criterion* defined by $Red _{\text {I}}(N) = \{\iota \mathbin \in Inf \mid concl (\iota ) \in N \}$ and $Red _{\text {F}}(N) = \emptyset$ clearly meets requirements (R1)–(R4). According to this criterion, an inference becomes redundant once its conclusion has been added to the set of formulas (e.g., by performing the inference), whereas formulas are never redundant.

#### Example 3

Assume $\models$ to be compact—i.e., $N \models \{C\}$ implies that $M \models \{C\}$ for some finite $M \subseteq N$.

Given a well-founded ordering $\succ$ on ${\mathbf {F}}$ and an inference system $Inf$ that satisfies $C_1 \succ C_0$ for all $(C_n,\ldots ,C_1,C_0) \in Inf$, the *standard redundancy criterion* defined by

$$\begin{aligned} Red _{\text {I}}(N)&:= \{(C_n,\ldots ,C_1,C_0) \in Inf \mid \{C_n,\ldots ,C_2\} \cup \{D \mathbin \in N \mid C_1 \succ D\} \models \{C_0\} \} \\ Red _{\text {F}}(N)&:= \{C \in {\mathbf {F}} \mid \{D \mathbin \in N \mid C \succ D\} \models \{C\} \} \end{aligned}$$

also meets requirements (R1)–(R4) [6, Sect. 4.2]. Intuitively, a formula is redundant if it is entailed by $\succ$-smaller formulas in *N*, whereas an inference is redundant if it is entailed by the side premises $C_n,\ldots ,C_2$ together with some formulas $M \subseteq N$ that are $\succ$-smaller than the main premise $C_1$. This criterion is used in conjunction with ordered resolution and superposition. Some authors require the subsets *M* to be finite but this is not necessary if $\models$ is compact. $Inf$ often satisfies $C_1 \succ C_k$ for all $k > 1$; the definition of $Red _{\text {I}}$ can then be simplified to $Red _{\text {I}}(N) := \{(C_n,\ldots ,C_1,C_0) \in Inf \mid \{D \mathbin \in N \mid C_1 \succ D\} \models \{C_0\} \}$.

#### Example 4

It is always possible to conflate redundant inferences and formulas by defining $Red _{\text {I}}$ such that $\iota \in Red _{\text {I}}(N)$ if and only if $\iota \in Inf$ and $concl (\iota ) \in N \cup Red _{\text {F}}(N)$ for all $\iota$ and *N*. This is, however, needlessly weak for many calculi. Consider superposition, which is based on the standard redundancy criterion. Let ${\mathsf {d}}> {\mathsf {c}}> {\mathsf {b}} > {\mathsf {a}}$ be the atom ordering, and assume the premises of the superposition inference 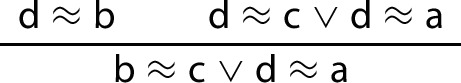 are in the clause set. Instead of performing the inference, we would like to exhaustively rewrite the second premise using the first premise, to ${\mathsf {b}} \approx {\mathsf {c}} \mathrel {\vee }{\mathsf {b}} \approx {\mathsf {a}}$, using either demodulation or a simultaneous superposition inference [11]. Notice that the conclusion does not become redundant in this way: It is entailed by ${\mathsf {b}} \approx {\mathsf {c}} \mathrel {\vee }{\mathsf {b}} \approx {\mathsf {a}}$ and ${\mathsf {d}} \approx {\mathsf {b}}$, but ${\mathsf {d}} \approx {\mathsf {b}}$ is $\succ$-larger than the conclusion and cannot be used. Nevertheless, with the standard redundancy criterion, the superposition inference becomes redundant, since the clause ${\mathsf {d}} \approx {\mathsf {b}}$ is smaller than the larger premise of the inference.

### Refutational Completeness

Let $\models$ be a consequence relation, let $Inf$ be an inference system, and let $Red$ be a redundancy criterion for $\models$ and $Inf$.

A set $N \subseteq {\mathbf {F}}$ is called *saturated* w.r.t. $Inf$ and $Red$ if $Inf (N) \subseteq Red _{\text {I}}(N)$. The pair $( Inf , Red )$ is called *statically refutationally complete* w.r.t. $\models$ if for every saturated set $N \subseteq {\mathbf {F}}$ such that $N \models \{\bot \}$ for some $\bot \in {\mathbf {F}}_\bot$, there exists a $\bot ' \in {\mathbf {F}}_\bot$ such that $\bot ' \in N$.

Let *A* be a set. An *A*-sequence is a finite sequence $(a_i)_{i=0}^k = a_0,a_1,\ldots ,a_k$ or an infinite sequence $(a_i)_{i=0}^\infty = a_0,a_1,\ldots$ with $a_i \in A$ for all indices *i*. We use the notation $(a_i)_{i\ge 0}$ or $(a_i)_i$ for both finite and infinite sequences. A nonempty sequence $(a_i)_i$ can be decomposed into a head $a_0$ and a tail $(a_i)_{i\ge 1}$. Given a relation ${\rhd } \subseteq A \times A$, a $\rhd$*-derivation* is a nonempty *A*-sequence such that $a_i \rhd a_{i+1}$ for all valid indices.

We define the relation ${\rhd _ Red } \subseteq \mathcal {P}({\mathbf {F}}) \times \mathcal {P}({\mathbf {F}})$ such that $N \rhd _ Red N'$ if and only if $N \setminus N' \subseteq Red _{\text {F}}(N')$.

In other words, when taking a transition, we may add arbitrary formulas ($N' \setminus N$) and remove redundant formulas ($N \setminus N'$). In practice, the added formulas would normally be entailed by *N*. But since our framework is designed to establish only dynamic refutational completeness, we impose no soundness restrictions on added formulas. If dynamic soundness of a prover is desired, it can be derived immediately from the soundness of the inferences and does not require its own framework.

Let $(N_i)_i$ be a $\mathcal {P}({\mathbf {F}})$-sequence. Its *limit* (*inferior*) is the set $N_\infty := \bigcup _i \bigcap _{j \ge i} N_{\!j}$. The limit consists of the *persistent formulas*: These are formulas that eventually emerge in some $N_i$ and remain present in all the following sets $N_{\!j}$ with $j \ge i$. The sequence is called (*weakly*) *fair* if $Inf (N_\infty ) \subseteq \bigcup _i Red _{\text {I}}(N_i)$. The pair $( Inf , Red )$ is called *dynamically refutationally complete* w.r.t. $\models$ if for every fair $\rhd _ Red$-derivation $(N_i)_i$ such that $N_0 \models \{\bot \}$ for some $\bot \in {\mathbf {F}}_\bot$, we have $\bot ' \in N_i$ for some *i* and some $\bot ' \in {\mathbf {F}}_\bot$.

#### Example 5

Let ${\Sigma }$ be a signature consisting of a binary predicate ${\mathsf {P}}$, a unary function ${\mathsf {f}}$, and a constant ${\mathsf {a}}$. Let $Inf$ be the set of inferences of the ordered resolution calculus with selection on $\Sigma$-clauses, and let $Red$ be the trivial redundancy criterion (Example 2). This calculus is refutationally complete [6, Sect. 3].

Consider the initial set $$N = \{{\mathsf {P}}(a,a), \lnot {\mathsf {P}}(x,x), {\mathsf {P}}({\mathsf {f}}(x),y)\vee \lnot {\mathsf {P}}(x,y)\}$$, where the second literal of the third clause is selected and no other literals are selected. The inference $$\iota = (\lnot {\mathsf {P}}(x,x),{\mathsf {P}}(a,a),\bot )$$ belongs to $$ Inf (N)$$ and derives $$\bot $$, so *N* is clearly unsatisfiable. However, without fairness, a derivation may never perform this inference. One such example is the infinite derivation

$$\begin{aligned} N \,\rhd _ Red \, N\cup \{{\mathsf {P}}({\mathsf {f}}({\mathsf {a}}),{\mathsf {a}})\} \,\rhd _ Red \, N\cup \{{\mathsf {P}}({\mathsf {f}}({\mathsf {a}}),{\mathsf {a}}), {\mathsf {P}}({\mathsf {f}}({\mathsf {f}}({\mathsf {a}})),{\mathsf {a}})\} \,\rhd _ Red \, \cdots \end{aligned}$$

where the clause $\lnot {\mathsf {P}}(x,x)$ is always ignored in favor of inferences between ${\mathsf {P}}({\mathsf {f}}(x),y)\vee \lnot {\mathsf {P}}(x,y)$ and a clause of the form ${\mathsf {P}}({\mathsf {f}}^i({\mathsf {a}}),{\mathsf {a}})$. This derivation is not fair because $\iota \in Inf (N_\infty )$, where $N_\infty = N \cup \{{\mathsf {P}}({\mathsf {f}}^i({\mathsf {a}}),{\mathsf {a}})\}_{i\in \mathbb {N}}$, but $\iota \notin \bigcup _i Red _{\text {I}}(N_i)$. In contrast, any derivation that is similar to the previous one but that performs the inference $\iota$ at some step would be fair.

Now consider the satisfiable set $N' = \{{\mathsf {P}}(a,a), {\mathsf {P}}({\mathsf {f}}(x),y)\vee \lnot {\mathsf {P}}(x,y)\}$. The derivation

$$\begin{aligned} N' \,\rhd _ Red \, N'\cup \{{\mathsf {P}}({\mathsf {f}}({\mathsf {a}}),{\mathsf {a}})\} \,\rhd _ Red \, N'\cup \{{\mathsf {P}}({\mathsf {f}}({\mathsf {a}}),{\mathsf {a}}), {\mathsf {P}}({\mathsf {f}}({\mathsf {f}}({\mathsf {a}})),{\mathsf {a}})\} \,\rhd _ Red \, \cdots \end{aligned}$$

is clearly fair.

Using properties (R1)–(R3), it is possible to show that static and dynamic refutational completeness agree [6]:

#### Lemma 6

If $(N_i)_i$ is a $\rhd _ Red$-derivation, then $(\bigcup _i {N}_i) \setminus N_\infty \subseteq Red _{\text {F}}(\bigcup _i {N}_i)$.

#### Proof

If $C \in (\bigcup _i {N}_i) \setminus N_\infty$, then there must exist some *i* such that $C \in N_i \setminus N_{i+1}$. Consequently, $C \in Red _{\text {F}}(N_{i+1})$. By property (R2), $C \in Red _{\text {F}}(\bigcup _i {N}_i)$. $\square$

#### Lemma 7

$(N_i)_i$ is a $\rhd _ Red$-derivation, then $Red _{\text {I}}(N_i) \subseteq Red _{\text {I}}(N_\infty )$ and $Red _{\text {F}}(N_i) \subseteq Red _{\text {F}}(N_\infty )$ for every *i*.

#### Proof

By property (R2), $Red _{\text {I}}(N_i) \subseteq Red _{\text {I}}(\bigcup _i {N}_i)$; by Lemma 6 and property (R3), $Red _{\text {I}}(\bigcup _i {N}_i) \subseteq Red _{\text {I}}((\bigcup _i {N}_i) \setminus ((\bigcup _i {N}_i) \setminus N_\infty )) = Red _{\text {I}}(N_\infty )$. Analogously, $Red _{\text {F}}(N_i) \subseteq Red _{\text {F}}(\bigcup _i {N}_i) \subseteq Red _{\text {F}}((\bigcup _i {N}_i) \setminus ((\bigcup _i {N}_i) \setminus N_\infty )) = Red _{\text {F}}(N_\infty )$. $\square$

#### Lemma 8

If $(N_i)_i$ is a $\rhd _ Red$-derivation, then $N_i \subseteq N_\infty \cup Red _{\text {F}}(N_\infty )$ for every *i*.

#### Proof

Let $C \in N_i$. If $C \notin N_\infty$, then there exists some $j \ge i$ such that $C \in N_{\!j} \setminus N_{j+1}$. Consequently, $C \in Red _{\text {F}}(N_{j+1})$ and therefore $C \in Red _{\text {F}}(N_\infty )$ by Lemma 7. $\square$

#### Lemma 9

If $(N_i)_i$ is a fair $\rhd _ Red$-derivation, then the limit $N_\infty$ is saturated w.r.t. $Inf$ and $Red$.

#### Proof

By fairness, every $\iota \in Inf (N_\infty )$ is contained in $\bigcup _i Red _{\text {I}}(N_i)$, so there exists some *i* such that $\iota \in Red _{\text {I}}(N_i)$, and thus $\iota \in Red _{\text {I}}(N_\infty )$ by Lemma 7. $\square$

Since fairness implies saturation of the limit, we could imagine simplifying the theory by defining dynamic refutational completeness in terms of derivations with saturated limits, instead of in terms of fair derivations. However, fairness more closely captures how provers work and is therefore easier to establish. A prover may delete formulas at different moments, without knowing the limit. This intuition is captured by the right-hand side $\bigcup _i Red _{\text {I}}(N_i)$ of the definition of fairness.

#### Lemma 10

If $( Inf , Red )$ is statically refutationally complete w.r.t. $\models$, then it is dynamically refutationally complete w.r.t. $\models$.

#### Proof

Assume $( Inf , Red )$ is statically refutationally complete w.r.t. $\models$, and let $(N_i)_i$ be a $\rhd _ Red$-derivation. Assume that $N_0 \models \{\bot \}$ for some $\bot \in {\mathbf {F}}_\bot$. Since $N_0 \subseteq \bigcup _i {N}_i$, we get $\bigcup _i {N}_i \models N_0 \models \{\bot \}$, and by property (R1), this implies $(\bigcup _i {N}_i) \setminus Red _{\text {F}}(\bigcup _i {N}_i) \models \{\bot \}$. By Lemma 6, we know that $(\bigcup _i {N}_i) \setminus N_\infty \subseteq Red _{\text {F}}(\bigcup _i {N}_i)$, or equivalently, $(\bigcup _i {N}_i) \setminus Red _{\text {F}}(\bigcup _i {N}_i) \subseteq N_\infty$; hence $N_\infty \models (\bigcup _i {N}_i) \setminus Red _{\text {F}}(\bigcup _i {N}_i) \models \{\bot \}$.

If the sequence is fair, then $N_\infty$ is saturated, so by static refutational completeness, $\bot ' \in N_\infty$ for some $\bot ' \in {\mathbf {F}}_\bot$. Consequently, $\bot ' \in N_i$ for some *i*, implying dynamic refutational completeness. $\square$

In fact, the converse holds as well:

#### Lemma 11

If $( Inf , Red )$ is dynamically refutationally complete w.r.t. $\models$, then it is statically refutationally complete w.r.t. $\models$.

#### Proof

Assume $( Inf , Red )$ is dynamically refutationally complete w.r.t. $\models$, and let $N_0 \subseteq {\mathbf {F}}$ be saturated w.r.t. $Inf$ and $Red$. Assume that $N_0 \models \bot$ for some $\bot \in {\mathbf {F}}_\bot$. Now consider the one-element sequence $(N_i)_{i=0}^0$. Since $N_\infty = N_0$ and $N_0$ is saturated, we know that $Inf (N_\infty ) = Inf (N_0) \subseteq Red _{\text {I}}(N_0) = \bigcup _i Red _{\text {I}}(N_i)$, so the sequence is fair. By dynamic refutational completeness, this implies $\bot ' \in N_0$ for some $\bot ' \in {\mathbf {F}}_\bot$. Therefore $( Inf , Red )$ is statically refutationally complete. $\square$

#### Example 12

Some popular preprocessing techniques, such as pure literal elimination and blocked clause elimination [26], are not covered by redundancy. These require inspecting the entire formula set and are not compatible with (R2). For example, ${\mathsf {P}}$ is pure in $\{{\mathsf {P}}\}$ but not in the larger set $\{{\mathsf {P}}, \lnot {\mathsf {P}}\}$.

In some situations, we may wish to apply techniques that delete clauses even if they are not redundant. If such techniques are used at most finitely many times, we can incorporate them in the framework by letting the initial set $N_0$ correspond to the set of formulas *after* all inprocessing has been performed, effectively considering all the transitions that happened before $N_0$ as one big preprocessing step. For example, a first-order prover might discover a clause that contains a pure predicate symbol (a predicate symbol that always occurs with the same polarity in the clause set) and delete it. If the signature is finite, this can be done only finitely many times and is hence compatible with the framework. We only need to show that inprocessing preserves unsatisfiability.

### Variations on a Theme

For some of the notions in Sects. 2.1 and 2.2 one can find alternative definitions in the literature.

#### Redundancy Criteria

As in Bachmair and Ganzinger’s chapter [6, Sect. 4.1], we have specified in condition (R1) of redundancy criteria that the deletion of redundant formulas must preserve inconsistency. Alternatively, one can require that redundant formulas must be entailed by the nonredundant ones—i.e., $N \setminus Red _{\text {F}}(N) \models Red _{\text {F}}(N)$—leading to some obvious changes in Lemmas 10 and 37.

Bachmair and Ganzinger’s definition of a redundancy criterion differs from ours in that they require only conditions (R1)–(R3). They call a redundancy criterion *effective* if an inference $\iota \in Inf$ is in $Red _{\text {I}}(N)$ whenever $concl (\iota ) \in N \cup Red _{\text {F}}(N)$. As demonstrated by Lemma 1, that condition is equivalent to our condition (R4).

#### Inferences from Redundant Premises

Inferences from redundant premises are sometimes excluded in the definitions of saturation, fairness, and refutational completeness  [6], and sometimes not  [5, 10, 30, 44].^1^ Similarly, redundancy of inferences is sometimes defined in such a way that inferences from redundant premises are necessarily redundant themselves  [5, 10], and sometimes not  [6, 30, 44]. There are good arguments for each of these choices. On the one hand, one can argue that the saturation of a set of formulas should not depend on the presence or absence of redundant formulas, and that inferences from redundant formulas should be redundant as well. On the other hand, in any reasonable proof system, formulas are deleted from the set of formulas as soon as they are shown to be redundant, so why should we care whether the set is saturated even if we do not delete formulas that have been proved to be redundant?

To clarify how the different definitions found in the literature relate to each other, we define “reduced” variants of the definitions in Sects. 2.1 and 2.2. A set $$N \subseteq {\mathbf {F}}$$ is called *reducedly saturated* w.r.t. $$ Inf $$ and $$ Red $$ if $$ Inf (N \setminus Red _{\text {F}}(N)) \subseteq Red _{\text {I}}(N)$$. The pair $$( Inf , Red )$$ is *reducedly statically refutationally complete* w.r.t. $$\models $$ if for every reducedly saturated set $$N \subseteq {\mathbf {F}}$$ with $$N \models \{\bot \}$$ for some $$\bot \in {\mathbf {F}}_\bot $$, there exists a $$\bot ' \in {\mathbf {F}}_\bot $$ such that $$\bot ' \in N$$. A sequence $$(N_i)_i$$ is called *reducedly fair* if $$ Inf (N_\infty \setminus \bigcup _i Red _{\text {F}}(N_i)) \subseteq \bigcup _i Red _{\text {I}}(N_i)$$. The pair $$( Inf , Red )$$ is *reducedly dynamically refutationally complete* w.r.t. $$\models $$ if for every reducedly fair $$\rhd _ Red $$-derivation $$(N_i)_i$$ such that $$N_0 \models \{\bot \}$$ for some $$\bot \in {\mathbf {F}}_\bot $$, we have $$\bot ' \in N_i$$ for some *i* and some $$\bot ' \in {\mathbf {F}}_\bot $$. A *reduced redundancy criterion* for $${\models }$$ and $$ Inf $$ is a redundancy criterion $$ Red = ( Red _{\text {I}}, Red _{\text {F}})$$ that additionally satisfies $$ Inf ({\mathbf {F}}, Red _{\text {F}}(N)) \subseteq Red _{\text {I}}(N)$$ for every $$N \subseteq {\mathbf {F}}$$. Recall that $$ Inf (N,M)$$ denotes the set of $$ Inf $$-inferences with at least one premise in *M* and the others in $$N \cup M$$.

For reduced redundancy criteria, saturation and reduced saturation agree:

##### Lemma 13

If $Red$ is a reduced redundancy criterion, then *N* is saturated w.r.t. $Inf$ and $Red$ if and only if *N* is reducedly saturated w.r.t. $Inf$ and $Red$.

##### Proof

If *N* is saturated w.r.t. $Inf$ and $Red$, then $Inf (N) \subseteq Red _{\text {I}}(N)$, so $Inf (N \setminus Red _{\text {F}}(N)) \subseteq Inf (N) \subseteq Red _{\text {I}}(N)$, which implies that *N* is reducedly saturated w.r.t. $Inf$ and $Red$.

Conversely, assume that *N* is reducedly saturated w.r.t. $Inf$ and $Red$—i.e., $Inf (N \setminus Red _{\text {F}}(N)) \subseteq Red _{\text {I}}(N)$. Let $\iota \in Inf (N)$. If no premise of $\iota$ is contained in $Red _{\text {F}}(N)$, then $\iota \in Inf (N \setminus Red _{\text {F}}(N)) \subseteq Red _{\text {I}}(N)$. Otherwise $\iota \in Inf ({\mathbf {F}}, Red _{\text {F}}(N))$, and since $Red$ is reduced, we get again $\iota \in Red _{\text {I}}(N)$. $\square$

##### Corollary 14

If $Red$ is a reduced redundancy criterion, then $( Inf , Red )$ is statically refutationally complete if and only if it is reducedly statically refutationally complete.

An arbitrary redundancy criterion $Red = ( Red _{\text {I}}, Red _{\text {F}})$ can always be extended to a reduced redundancy criterion $Red ' = ( Red _{\text {I}}', Red _{\text {F}})$, where $Red _{\text {I}}'$ is defined by $Red _{\text {I}}'(N) := Red _{\text {I}}(N) \cup Inf ({\mathbf {F}}, Red _{\text {F}}(N))$ for all *N*.

##### Lemma 15

$$ Red '$$ is a reduced redundancy criterion.

##### Proof

Since $$ Red _{\text {F}}$$ is left unchanged, (R1) and the first parts of (R2) and (R3) are obvious. (R4) holds because $$\iota \in Red _{\text {I}}(N) \subseteq Red _{\text {I}}'(N)$$ for every inference $$\iota $$ with $$ concl (\iota ) \in N$$. Moreover, $$ Red '$$ is clearly reduced. It remains to prove the second parts of (R2) and (R3).

For (R2), assume $N \subseteq N'$. Then $Red _{\text {I}}(N) \subseteq Red _{\text {I}}(N')$ and $Red _{\text {F}}(N) \subseteq Red _{\text {F}}(N')$. Moreover, $Inf$ is clearly monotonic, so $Inf ({\mathbf {F}}, Red _{\text {F}}(N)) \subseteq Inf ({\mathbf {F}}, Red _{\text {F}}(N'))$, and therefore $Red _{\text {I}}'(N) \subseteq Red _{\text {I}}'(N')$.

For (R3), assume $N' \subseteq Red _{\text {F}}(N)$. Then $Red _{\text {F}}(N) \subseteq Red _{\text {F}}(N \setminus N')$ and $Red _{\text {I}}(N) \subseteq Red _{\text {I}}(N \setminus N')$. By monotonicity of $Inf$, we have $Inf ({\mathbf {F}}, Red _{\text {F}}(N)) \subseteq Inf ({\mathbf {F}}, Red _{\text {F}}(N \setminus N'))$, so $Red _{\text {I}}'(N) \subseteq Red _{\text {I}}'(N \setminus N')$. $\square$

##### Lemma 16

If $$N \subseteq {\mathbf {F}}$$ is saturated w.r.t. $$ Inf $$ and $$ Red $$, then *N* is saturated w.r.t. $$ Inf $$ and $$ Red '$$.

##### Proof

Since $$ Red _{\text {I}}(N) \subseteq Red _{\text {I}}'(N)$$, $$ Inf (N) \subseteq Red _{\text {I}}(N)$$ implies $$ Inf (N) \subseteq Red _{\text {I}}'(N)$$. $$\square $$

The converse does not hold:

##### Example 17

Consider a signature consisting of the four propositional variables (or nullary predicate symbols) ${\mathsf {P}}$, ${\mathsf {Q}}$, ${\mathsf {R}}$, ${\mathsf {S}}$. Let $Inf$ be the set of inferences of the ordered resolution calculus with selection over clauses over the signature. Define $Red _{\text {F}}$ such that a clause *C* is contained in $Red _{\text {F}}(N)$ if it is entailed by clauses in *N* that are smaller than *C*. Define $Red _{\text {I}}$ such that an inference is contained in $Red _{\text {I}}(N)$ if its conclusion is entailed by clauses in *N* that are smaller than its largest premise. Then $Red := ( Red _{\text {I}}, Red _{\text {F}})$ is a redundancy criterion.

Let *N* be the set of clauses (1) $$\lnot {\mathsf {Q}} \vee {\mathsf {P}}$$, (2) $$\lnot {\mathsf {S}} \vee {\mathsf {R}} \vee {\mathsf {Q}}$$, (3) $$\lnot {\mathsf {S}} \vee {\mathsf {Q}}$$, where the atom ordering is $${\mathsf {P}}> {\mathsf {Q}}> {\mathsf {R}} > {\mathsf {S}}$$ and the first literals of (1) and (3) are selected. Due to the selection, $$ Inf (N)$$ contains only a single inference, namely the ordered resolution inference $$\iota $$ between (2) and (1). The largest premise of $$\iota $$ is (1). The premise (2) is entailed by the smaller clause (3) and therefore contained in $$ Red _{\text {F}}(N)$$. Consequently, $$\iota \in Red _{\text {I}}'(N)$$, which means that *N* is saturated w.r.t. $$ Red '$$. On the other hand, the conclusion $$\lnot {\mathsf {S}} \mathrel {\vee }{\mathsf {R}} \mathrel {\vee }{\mathsf {P}}$$ is not entailed by the clauses in *N* that are smaller than (1)—i.e., (2) and (3)—so $$\iota \notin Red _{\text {I}}(N)$$. Therefore, *N* is not saturated w.r.t. $$ Red $$.

##### Lemma 18

The following properties are equivalent for every $N \subseteq {\mathbf {F}}$:

(i) *N* is reducedly saturated w.r.t. $Inf$ and $Red$;
(ii) *N* is saturated w.r.t. $$ Inf $$ and $$ Red '$$;
(iii) $N \setminus Red _{\text {F}}(N)$ is saturated w.r.t. $Inf$ and $Red$.

##### Proof

To show that (i) implies (ii), assume that *N* is reducedly saturated w.r.t. $Inf$ and $Red$—i.e., $Inf (N \setminus Red _{\text {F}}(N)) \subseteq Red _{\text {I}}(N)$. We must show that $Inf (N) \subseteq Red _{\text {I}}'(N)$. Let $\iota \in Inf (N)$. If no premise of $\iota$ is contained in $Red _{\text {F}}(N)$, then $\iota \in Inf (N \setminus Red _{\text {F}}(N)) \subseteq Red _{\text {I}}(N)$. Otherwise, $\iota \in Inf ({\mathbf {F}}, Red _{\text {F}}(N))$. In both cases, we conclude $\iota \in Red _{\text {I}}'(N)$.

To show that (ii) implies (i), assume that *N* is saturated w.r.t. $$ Inf $$ and $$ Red '$$—i.e., $$ Inf (N) \subseteq Red _{\text {I}}'(N)$$. We must show that $$ Inf (N \setminus Red _{\text {F}}(N)) \subseteq Red _{\text {I}}(N)$$. Let $$\iota \in Inf (N \setminus Red _{\text {F}}(N))$$. Observe first that $$\iota \in Inf (N \setminus Red _{\text {F}}(N)) \subseteq Inf (N) \subseteq Red _{\text {I}}'(N) = Red _{\text {I}}(N) \cup Inf ({\mathbf {F}}, Red _{\text {F}}(N))$$. Moreover, $$\iota \in Inf (N \setminus Red _{\text {F}}(N))$$ implies $$\iota \notin Inf ({\mathbf {F}}, Red _{\text {F}}(N))$$. Combining both, we get $$\iota \in Red _{\text {I}}(N)$$.

The equivalence of (i)—i.e., $Inf (N \setminus Red _{\text {F}}(N)) \subseteq Red _{\text {I}}(N)$—and (iii)—i.e., $Inf (N \setminus Red _{\text {F}}(N)) \subseteq Red _{\text {I}}(N \setminus Red _{\text {F}}(N))$—follows from the fact that $Red _{\text {I}}(N) \subseteq Red _{\text {I}}(N \setminus Red _{\text {F}}(N))$ by (R3) and $Red _{\text {I}}(N \setminus Red _{\text {F}}(N)) \subseteq Red _{\text {I}}(N)$ by (R2). $\square$

Even though $$ Red $$ and $$ Red '$$ are not equivalent as far as saturation is concerned, they are equivalent w.r.t. refutational completeness:

##### Theorem 19

The following properties are equivalent:

(i) $( Inf , Red )$ is statically refutationally complete w.r.t. $\models$;
(ii) $( Inf , Red )$ is reducedly statically refutationally complete w.r.t. $\models$;
(iii) $( Inf , Red ')$ is statically refutationally complete w.r.t. $\models$;
(iv) $( Inf , Red ')$ is reducedly statically refutationally complete w.r.t. $\models$.

##### Proof

To show that (iii) implies (i), assume that $( Inf , Red ')$ is statically refutationally complete. That is, the property

$$\begin{aligned} N \models \{\bot \}\,\text { for some}\, \bot \in {\mathbf {F}}_\bot \,\text { implies}\, \bot ' \in N\,\text { for some }\,\bot ' \in {\mathbf {F}}_\bot \quad \quad \quad \quad \quad \quad \quad (*) \end{aligned}$$

holds for every set $$N \subseteq {\mathbf {F}}$$ that is saturated w.r.t. $$ Inf $$ and $$ Red '$$. By Lemma 16, every set $$N \subseteq {\mathbf {F}}$$ that is saturated w.r.t. $$ Inf $$ and $$ Red $$ is also saturated w.r.t. $$ Inf $$ and $$ Red '$$, so property $$(*)$$ holds in particular for every set $$N \subseteq {\mathbf {F}}$$ that is saturated w.r.t. $$ Inf $$ and $$ Red $$.

To show that (i) implies (iii), assume that $$( Inf , Red )$$ is statically refutationally complete. Assume *N* is saturated w.r.t. $$ Inf $$ and $$ Red '$$ and suppose that $$N \models \{\bot \}$$ for some $$\bot \in {\mathbf {F}}_\bot $$. By Lemma 18, $$N \setminus Red _{\text {F}}(N)$$ is saturated w.r.t. $$ Inf $$ and $$ Red $$. Furthermore, by (R1), $$N \setminus Red _{\text {F}}(N) \models \{\bot \}$$. So the static refutational completeness of $$( Inf , Red )$$ implies that $$\bot ' \in N \setminus Red _{\text {F}}(N)$$ for some $$\bot ' \in {\mathbf {F}}_\bot $$; hence $$\bot ' \in N$$. Thus, $$( Inf , Red ')$$ is statically refutationally complete.

The equivalence of (iii) and (iv) follows from Lemma 15 and Corollary 14.

It remains to show the equivalence of (ii) and (iii). Observe that (ii) means that $$(*)$$ holds for every set $$N \subseteq {\mathbf {F}}$$ that is reducedly saturated w.r.t. $$ Inf $$ and $$ Red $$, and that (iii) means that $$(*)$$ holds for every set $$N \subseteq {\mathbf {F}}$$ that is saturated w.r.t. $$ Inf $$ and $$ Red '$$. By Lemma 18, these two properties are equivalent. $$\square $$

The limit of a reducedly fair $\rhd _ Red$-derivation is a reducedly saturated set.^2^ This is proved analogously to Lemma 9:

##### Lemma 20

If $(N_i)_i$ is a reducedly fair $\rhd _ Red$-derivation, then the limit $N_\infty$ is reducedly saturated w.r.t. $Inf$ and $Red$.

##### Proof

Since $Red _{\text {F}}(N_i) \subseteq Red _{\text {F}}(N_\infty )$ for every *i*, we have $Inf (N_\infty \setminus Red _{\text {F}}(N_\infty )) \subseteq Inf (N_\infty \setminus \bigcup _i Red _{\text {F}}(N_i))$. By reduced fairness, every inference $\iota \in Inf (N_\infty \setminus Red _{\text {F}}(N_\infty ))$ is contained in $\bigcup _i Red _{\text {I}}(N_i)$. Therefore there exists some *i* with $\iota \in Red _{\text {I}}(N_i)$, implying $\iota \in Red _{\text {I}}(N_\infty )$. $\square$

Lemmas 10 and 11 can then be reproved for reduced static and reduced dynamic refutational completeness. Together with Theorem 19, we obtain this result:

##### Theorem 21

The properties (i)–(iv) of Theorem 19 and the following four properties are equivalent:

(v) $( Inf , Red )$ is dynamically refutationally complete w.r.t. $\models$;
(vi) $( Inf , Red )$ is reducedly dynamically refutationally complete w.r.t. $\models$;
(vii) $( Inf , Red ')$ is dynamically refutationally complete w.r.t. $\models$;
(viii) $( Inf , Red ')$ is reducedly dynamically refutationally complete w.r.t. $\models$.

Summarizing, we see that there are some differences between the “reduced” and the “nonreduced” approach, but that these differences are restricted to the intermediate notions, notably saturation. As far as (static or dynamic) refutational completeness is concerned, both approaches agree. Furthermore, Theorem 21 demonstrates that we can mix and match definitions from both worlds. Consequently, when we want to build on an existing refutational completeness proof for some saturation calculus, it does not matter which approach has been used there.

Given that the “nonreduced” definitions in Sects. 2.1 and 2.2 are simpler that than the “reduced” ones in the current section, there is usually little reason to prefer the “reduced” ones. For our purposes, a major advantage of the “nonreduced” definitions is that $Red _{\text {F}}$ and $Red _{\text {I}}$ are separated as much as possible. In particular, our definitions of saturation and static refutational completeness do not depend on redundant formulas, but only on redundant inferences. This property will be crucial for the proof of Theorem 45 in Sect. 3.

#### Fairness in the Limit

Bachmair and Ganzinger consider a sequence $(N_i)_i$ fair if $concl ( Inf (N') \setminus Red _{\text {I}}(N')) \subseteq (\bigcup _i {N}_i) \cup Red _{\text {F}}(\bigcup _i {N}_i)$, where $N' = N_\infty \setminus Red _{\text {F}}(N_\infty )$ [6, Sect. 4.1]. This is a quite peculiar property. First of all, it is overly complicated: If the conclusion of an inference $\iota \in Inf (N') \setminus Red _{\text {I}}(N')$ is contained in $(\bigcup _i {N}_i) \cup Red _{\text {F}}(\bigcup _i {N}_i)$, then $\iota \in Red _{\text {I}}(\bigcup _i {N}_i)$ by Lemma 1 and (R4), and by Lemma 6 and (R3) we have $\iota \in Red _{\text {I}}(\bigcup _i {N}_i) \subseteq Red _{\text {I}}((\bigcup _i {N}_i) \setminus ((\bigcup _i {N}_i) \setminus N_\infty )) = Red _{\text {I}}(N_\infty ) \subseteq Red _{\text {I}}(N_\infty \setminus Red _{\text {F}}(N_\infty )) = Red _{\text {I}}(N')$. But this contradicts the assumption that $\iota \in Inf (N') \setminus Red _{\text {I}}(N')$. So the condition can be simplified to $Inf (N') \subseteq Red _{\text {I}}(N')$, and since $Red _{\text {I}}(N') = Red _{\text {I}}(N_\infty \setminus Red _{\text {F}}(N_\infty )) = Red _{\text {I}}(N_\infty )$, this is equivalent to $Inf (N_\infty \setminus Red _{\text {F}}(N_\infty )) \subseteq Red _{\text {I}}(N_\infty )$.

Since $Inf (N_\infty \setminus Red _{\text {F}}(N_\infty )) \subseteq Inf (N_\infty \setminus \bigcup _i Red _{\text {F}}(N_i))$ and $\bigcup _i Red _{\text {I}}(N_i) \subseteq Red _{\text {I}}(N_\infty )$, the (simplified) condition is entailed by reduced fairness. There is a crucial difference, though: While reduced fairness requires that every inference from $N_\infty$ is redundant or has a redundant premise at some finite step of the derivation, the Bachmair–Ganzinger definition also admits derivations where redundancy is achieved only in the limit.

##### Example 22

Consider a signature consisting of two unary predicate symbols ${\mathsf {P}}$, ${\mathsf {Q}}$, a unary function symbol ${\mathsf {f}}$, and a constant ${\mathsf {b}}$. Let $Inf$ be the set of inferences of the ordered resolution calculus with selection over clauses over the signature.

Let *N* be the set of clauses (1) ${\mathsf {P}}({\mathsf {b}})$, (2) $\lnot {\mathsf {P}}(x) \mathrel {\vee }{\mathsf {P}}({\mathsf {f}}(x))$, (3) ${\mathsf {Q}}({\mathsf {b}})$, (4) $\lnot {\mathsf {Q}}({\mathsf {b}}) \mathrel {\vee }{\mathsf {P}}({\mathsf {f}}(x))$, where the atom ordering is a lexicographic path ordering with precedence ${\mathsf {P}}> {\mathsf {Q}}> {\mathsf {f}} > {\mathsf {b}}$ and the first literals of (2) and (4) are selected. From (1) and (2), we obtain in the first derivation step ${\mathsf {P}}({\mathsf {f}}({\mathsf {b}}))$, in the second step ${\mathsf {P}}({\mathsf {f}}({\mathsf {f}}({\mathsf {b}})))$, and so on. The limit $N_\infty$ consists of the four initial clauses (1)–(4) and all clauses of the form ${\mathsf {P}}({\mathsf {f}}^i({\mathsf {b}}))$ with $i \ge 1$. The resolution inference between (3) and (4), yielding ${\mathsf {P}}({\mathsf {f}}(x))$, is therefore redundant w.r.t. $N_\infty$, since for each of its ground instances the conclusion ${\mathsf {P}}({\mathsf {f}}^i({\mathsf {b}}))$ is contained in $N_\infty$. However, it is not redundant w.r.t. any set $N_{\!j}$. Similarly, the premise (4) is redundant w.r.t. $N_\infty$ but not w.r.t. any set $N_{\!j}$. Therefore, the sequence of clause sets is fair according to the definition in Bachmair and Ganzinger [6, Sect. 4.1], but neither fair nor reducedly fair according to our definitions.

Of course, a redundancy property that holds only for the limit of an infinite sequence generally cannot be checked effectively. In other words, Bachmair and Ganzinger’s definition is more permissive than our alternative definition, but the additional degree of freedom can hardly be exploited in a theorem prover.

#### Semi-redundancy

Bachmair, Ganzinger, and Waldmann [8] use a definition of redundancy criteria that requires (R2) only for formulas and (R3) only for inferences. With their definition of fairness, this is sufficient to show that the limit of a fair $\rhd _ Red$-derivation is saturated, and thus, to show that static refutational completeness implies dynamic refutational completeness. Their definition of fairness, however, requires essentially that inferences from formulas in the limit $N_\infty$ are redundant w.r.t. the limit, and since they do not enforce that an inference that is redundant at some step of the derivation is redundant w.r.t. the limit, this cannot be checked effectively in a theorem prover.

#### Nonstrict Redundancy

Nieuwenhuis and Rubio [29, 30] and Peltier [34] define a ground clause *C* to be nonstrictly redundant w.r.t. a set *N* of ground clauses if *C* is entailed by smaller *or equal* clauses in *N*. This definition does not satisfy our condition (R3). Consequently, it can be used for proving the static completeness of a calculus, but it is insufficient to establish the connection between static and dynamic completeness (unless the notion of fairness is strengthened).

### Intersections of Redundancy Criteria

In descriptions of concrete saturation calculi, we frequently encounter the situation that the calculus is parameterized in some way and that exactly one value of the parameter is used to show that every saturated $N \subseteq {\mathbf {F}}$ with $N \models \{\bot \}$ contains $\bot$, but that this value is still unknown during the actual saturation process. Consequently, inferences and formulas may be considered as redundant during the saturation only if they are redundant for every possible value of the parameter. To model this situation in our framework, it is useful to define consequence relations and redundancy criteria as intersections of previously defined consequence relations or redundancy criteria.

Let *Q* be an arbitrary nonempty set, and let $({\models ^q})_{q \in Q}$ be a *Q*-indexed family of consequence relations over ${\mathbf {F}}$. Define ${\models }^\cap := \bigcap _{q \in Q} {\models ^q}$.

#### Lemma 23

${\models }^\cap$ is a consequence relation.

#### Proof

Obvious. $$\square $$

Let $Inf$ be an inference system, and let $( Red ^q)_{q \in Q}$ be a *Q*-indexed family of redundancy criteria, where each $Red ^q = ( Red _{\text {I}}^q, Red _{\text {F}}^q)$ is a redundancy criterion for $Inf$ and $\models ^q$. Let $Red _{\text {I}}^\cap (N) := \bigcap _{q \in Q} Red _{\text {I}}^q(N)$ and $Red _{\text {F}}^\cap (N) := \bigcap _{q \in Q} Red _{\text {F}}^q(N)$ for all *N*. Define $Red ^\cap := ( Red _{\text {I}}^\cap , Red _{\text {F}}^\cap )$.

#### Lemma 24

$Red ^\cap$ is a redundancy criterion for ${\models }^\cap$ and $Inf$.

#### Proof

(R1) Assume that $$N \models ^\cap _{\mathcal {G}}\{\bot \}$$ for some $$\bot \in {\mathbf {F}}_\bot $$—i.e., $$N \models ^q \{\bot \}$$ for every $$q \in Q$$. As $$ Red _{\text {F}}^\cap (N) \subseteq Red _{\text {F}}^q(N)$$, we have $$N \setminus Red _{\text {F}}^\cap (N) \supseteq N \setminus Red _{\text {F}}^q(N)$$, and by (C2) $$N \setminus Red _{\text {F}}^\cap (N) \models ^q N \setminus Red _{\text {F}}^q(N)$$. Furthermore, $$N \setminus Red _{\text {F}}^q(N) \models ^q \{\bot \}$$ by (R1) for $$ Red ^q$$. So $$N \setminus Red _{\text {F}}^\cap (N) \models ^q \{\bot \}$$ by (C4) for every $$q \in Q$$ and therefore $$N \setminus Red _{\text {F}}(N) \models ^\cap _{\mathcal {G}}\{\bot \}$$.

(R2) Let $N \subseteq N'$. Since $Red _{\text {F}}^q(N) \subseteq Red _{\text {F}}^q(N')$ for every *q*, we have $Red _{\text {F}}^\cap (N) = \bigcap _{q \in Q} Red _{\text {F}}^q(N) \subseteq \bigcap _{q \in Q} Red _{\text {F}}^q(N') = Red _{\text {F}}^\cap (N')$ and analogously for $Red _{\text {I}}^\cap$.

(R3) Let $N' \subseteq Red _{\text {F}}(N)$. Since $Red _{\text {F}}^q(N) \subseteq Red _{\text {F}}^q(N \setminus N')$ for every *q*, we have $Red _{\text {F}}^\cap (N) = \bigcap _{q \in Q} Red _{\text {F}}^q(N) \subseteq \bigcap _{q \in Q} Red _{\text {F}}^q(N \setminus N') = Red _{\text {F}}^\cap (N \setminus N')$ and analogously for $Red _{\text {I}}^\cap$.

(R4) If $\iota \in Inf$ and $concl (\iota ) \in N$, then $\iota \in Red _{\text {I}}^q(N)$ for every $q \in Q$; hence $\iota \in \bigcap _{q \in Q} Red _{\text {I}}^q(N) = Red _{\text {I}}^\cap (N)$. $\square$

#### Lemma 25

A set $$N \subseteq {\mathbf {F}}$$ is saturated w.r.t. $$ Inf $$ and $$ Red ^\cap $$ if and only if it is saturated w.r.t. $$ Inf $$ and $$ Red ^q$$ for every $$q \in Q$$.

#### Proof

If *N* is saturated w.r.t. $$ Inf $$ and $$ Red ^\cap $$, then $$ Inf (N) \subseteq Red _{\text {I}}^\cap (N) = \bigcap _{q \in Q} Red _{\text {I}}^q(N)$$; hence $$ Inf (N) \subseteq Red _{\text {I}}^q(N)$$ for every $$q \in Q$$, implying that *N* is saturated w.r.t. $$ Inf $$ and $$ Red ^q$$.

Conversely, if *N* is saturated w.r.t. $$ Inf $$ and $$ Red ^q$$ for every $$q \in Q$$, then $$ Inf (N) \subseteq Red _{\text {I}}^q(N)$$ for every $$q \in Q$$; hence $$ Inf (N) \subseteq Red _{\text {I}}^\cap (N) = \bigcap _{q \in Q} Red _{\text {I}}^q(N)$$, which implies that *N* is saturated w.r.t. $$ Inf $$ and $$ Red ^\cap $$. $$\square $$

In many cases where a redundancy criterion $Red ^\cap$ is defined as the intersection of other criteria, the consequence relations ${\models ^q}$ agree for all $q \in Q$.

There are some exceptions, though, for example constraint superposition [29], where the parameter *q* is a convergent rewrite system *R* and ${\models ^q}$ is entailment modulo *R*. For such calculi, one can typically demonstrate the static refutational completeness of $( Inf , Red ^\cap )$ in the following form:

#### Lemma 26

If for every set $N \subseteq {\mathbf {F}}$ that is saturated w.r.t. $Inf$ and $Red ^\cap$ and does not contain any $\bot ' \in {\mathbf {F}}_\bot$ there exists some $q \in Q$ such that $N \not \models ^q \{\bot \}$ for some $\bot \in {\mathbf {F}}_\bot$, then $( Inf , Red ^\cap )$ is statically refutationally complete w.r.t. ${\models ^\cap }$.

#### Proof

Suppose that $N \subseteq {\mathbf {F}}$ is saturated w.r.t. $Inf$ and $Red ^\cap$ and $N \models ^\cap \{\bot '\}$ for some $\bot ' \in {\mathbf {F}}_\bot$. Consequently, $N \models ^q \{\bot '\}$ for every $q \in Q$. By (C1), $N \models ^q \{\bot '\} \models ^q \{\bot \}$ for every $\bot \in {\mathbf {F}}_\bot$. If the condition of the lemma holds, then *N* must contain some $\bot '' \in {\mathbf {F}}_\bot$. Therefore, $( Inf , Red ^\cap )$ is statically refutationally complete w.r.t. ${\models ^\cap }$. $\square$

## Lifting

A standard approach for establishing the refutational completeness of a calculus is to first concentrate on the ground case and then lift the results to the nonground case. In this section, we show how to perform this lifting abstractly, given a suitable grounding function ${\mathcal {G}}$. The function maps every formula $C \in {\mathbf {F}}$ to a set ${\mathcal {G}}(C)$ of formulas from a set of formulas ${\mathbf {G}}$. Depending on the logic and the calculus, ${\mathcal {G}}(C)$ may be, for example, the set of all ground instances of *C*, a subset of the set of ground instances of *C*, or even a set of formulas from another logic. Similarly, $FInf$-inferences are mapped to sets of $GInf$-inferences, and saturation w.r.t. $FInf$-inferences is related to saturation w.r.t. $GInf$-inferences.

There are calculi where some $FInf$-inferences $\iota$ do not have a counterpart in $GInf$, such as the PosExt inferences of $\lambda$-free superposition [12]. In these cases, we set ${\mathcal {G}}(\iota ) = undef$.

### Standard Lifting

Given two sets of formulas ${\mathbf {F}}$ and ${\mathbf {G}}$, an ${\mathbf {F}}$-inference system $FInf$, a ${\mathbf {G}}$-inference system $GInf$, and a redundancy criterion $Red$ for $GInf$, let ${\mathcal {G}}$ be a function that maps every formula in ${\mathbf {F}}$ to a subset of ${\mathbf {G}}$ and every ${\mathbf {F}}$-inference in $FInf$ to $undef$ or to a subset of $GInf$. The function ${\mathcal {G}}$ is called a *grounding function* if

1. for every $$\bot \in {\mathbf {F}}_\bot $$, $$\emptyset \not = {\mathcal {G}}(\bot ) \subseteq {\mathbf {G}}_\bot $$;
2. for every $C \in {\mathbf {F}}$, if $\bot \in {\mathcal {G}}(C)$ and $\bot \in {\mathbf {G}}_\bot$ then $C \in {\mathbf {F}}_\bot$;
3. for every $\iota \in FInf$, if ${\mathcal {G}}(\iota ) \not = undef$, then ${\mathcal {G}}(\iota ) \subseteq Red _{\text {I}}({\mathcal {G}}( concl (\iota )))$.

The function ${\mathcal {G}}$ is extended to sets $N \subseteq {\mathbf {F}}$ by defining ${\mathcal {G}}(N) := \bigcup _{C \in N} {\mathcal {G}}(C)$ for all *N*. Analogously, for a set $I \subseteq FInf$, ${\mathcal {G}}(I) := \bigcup _{\iota \in I,\, {\mathcal {G}}(\iota ) \not = undef } {\mathcal {G}}(\iota )$.

#### Remark 27

Conditions (G1) and (G2) express that *false* formulas may only be mapped to sets of *false* formulas, and that only *false* formulas may be mapped to sets of *false* formulas. For most applications, it would be possible to replace condition (G3) by

$$(\mathrm{G}3')$$
for every $\iota \in FInf$, if ${\mathcal {G}}(\iota ) \not = undef$, then $concl ({\mathcal {G}}(\iota )) \subseteq {\mathcal {G}}( concl (\iota ))$,

which implies (G3) by property (R4). There are some calculi, however, for which (G3$'$) is too strong. Typical examples are calculi where the ${\mathbf {F}}$-inferences include some normalization or abstraction step that does not have a counterpart in the ${\mathbf {G}}$-inferences. So an ${\mathbf {F}}$-inference $\iota$ may have a conclusion $C \mathrel {\vee }t \not \approx t'$, where the literal $t \not \approx t'$ results from the normalization step, but the conclusions of the instances of $\iota$ have the form $C\theta$ for a substitution $\theta$ that unifies *t* and $t'$. In this case, (G3) is still satisfied, but (G3$'$) is not.

#### Example 28

In standard superposition, ${\mathbf {F}}$ is the set of all universally quantified first-order clauses over some signature $\Sigma$, ${\mathbf {G}}$ is the set of all ground first-order clauses over $\Sigma$, and ${\mathcal {G}}$ maps every clause *C* to the set of its ground instances $C\theta$ and every superposition inference $\iota$ to the set of its ground instances $\iota \theta$.

Let ${\mathcal {G}}$ be a grounding function from ${\mathbf {F}}$ and $FInf$ to ${\mathbf {G}}$ and $GInf$, and let ${\models } \subseteq \mathcal {P}({\mathbf {G}}) \times \mathcal {P}({\mathbf {G}})$ be a consequence relation over ${\mathbf {G}}$. We define the relation ${\models _{\mathcal {G}}} \subseteq \mathcal {P}({\mathbf {F}}) \times \mathcal {P}({\mathbf {F}})$ such that $N_1 \models _{\mathcal {G}}N_2$ if and only if ${\mathcal {G}}(N_1) \models {\mathcal {G}}(N_2)$. We call ${\models _{\mathcal {G}}}$ the ${\mathcal {G}}$-*lifting* of ${\models }$. It corresponds to Herbrand entailment. If Tarski entailment (i.e., $N_1 \models _\text {T} N_2$ if and only if any model of $N_1$ is also a model of $N_2$) is desired, the mismatch can be repaired by showing that the two notions of entailment are equivalent as far as refutations are concerned.

#### Lemma 29

$\models _{\mathcal {G}}$ is a consequence relation over ${\mathbf {F}}$.

#### Proof

(C1) Let $\bot \in {\mathbf {F}}_\bot$. Then by property (G1) of grounding functions, ${\mathcal {G}}(\{\bot \})$ contains some $\bot ' \in {\mathbf {G}}_\bot$. So ${\mathcal {G}}(\{\bot \}) \models \{\bot '\} \models {\mathcal {G}}(N_1)$ for every $N_1$, and hence $\{\bot \} \models _{\mathcal {G}}N_1$ as required.

(C2) Let $N_2 \subseteq N_1$. Then ${\mathcal {G}}(N_2) \subseteq {\mathcal {G}}(N_1)$, so ${\mathcal {G}}(N_1) \models {\mathcal {G}}(N_2)$, and thus $N_1 \models _{\mathcal {G}}N_2$.

(C3) Suppose that $N_1 \models _{\mathcal {G}}\{C\}$ for every $C \in N_2$. Then ${\mathcal {G}}(N_1) \models {\mathcal {G}}(\{C\})$ for every $C \in N_2$ and therefore ${\mathcal {G}}(N_1) \models \bigcup _{C \in N_2} {\mathcal {G}}(\{C\}) = {\mathcal {G}}(N_2)$; hence $N_1 \models _{\mathcal {G}}N_2$.

(C4) Suppose that $N_1 \models _{\mathcal {G}}N_2$ and $N_2 \models _{\mathcal {G}}N_3$. Then ${\mathcal {G}}(N_1) \models {\mathcal {G}}(N_2)$ and ${\mathcal {G}}(N_2) \models {\mathcal {G}}(N_3)$; therefore ${\mathcal {G}}(N_1) \models {\mathcal {G}}(N_3)$, and therefore $N_1 \models _{\mathcal {G}}N_3$. $\square$

Let $Red = ( Red _{\text {I}}, Red _{\text {F}})$ be a redundancy criterion for $\models$ and $GInf$. We define functions $Red _{\text {I}}^{\mathcal {G}}: \mathcal {P}({\mathbf {F}}) \rightarrow \mathcal {P}( FInf )$ and $Red _{\text {F}}^{\mathcal {G}}: \mathcal {P}({\mathbf {F}}) \rightarrow \mathcal {P}({\mathbf {F}})$ by

$$\begin{aligned} \begin{array}{@{}l@{}} \iota \in Red _{\text {I}}^{\mathcal {G}}(N)~~\text {if and only if}~~\\ \quad {\mathcal {G}}(\iota ) \not = undef \, \text {and}\, {\mathcal {G}}(\iota ) \subseteq Red _{\text {I}}({\mathcal {G}}(N))\\ \quad \text {or}\, {\mathcal {G}}(\iota ) = undef \, \text {and}\, {\mathcal {G}}( concl (\iota )) \subseteq {\mathcal {G}}(N) \cup Red _{\text {F}}({\mathcal {G}}(N));\\ C \in Red _{\text {F}}^{\mathcal {G}}(N)~~\text {if and only if}~~\\ \quad {\mathcal {G}}(C) \subseteq Red _{\text {F}}({\mathcal {G}}(N)). \end{array} \end{aligned}$$

We call $Red ^{\mathcal {G}}:= ( Red _{\text {I}}^{\mathcal {G}}, Red _{\text {F}}^{\mathcal {G}})$ the ${\mathcal {G}}$-*lifting* of $Red$.

#### Theorem 30

$Red ^{\mathcal {G}}$ is a redundancy criterion for $\models _{\mathcal {G}}$ and $FInf$.

We omit the proof at this point since we will prove a more general result (Theorem 42) in Sect. 3.2. The following folklore lemma connects a nonground calculus with a ground calculus it overapproximates.

#### Lemma 31

If $N \subseteq {\mathbf {F}}$ is saturated w.r.t. $FInf$ and $Red ^{\mathcal {G}}$ and $GInf ({\mathcal {G}}(N)) \subseteq {\mathcal {G}}( FInf (N)) \cup Red _{\text {I}}({\mathcal {G}}(N))$, then ${\mathcal {G}}(N)$ is saturated w.r.t. $GInf$ and $Red$.

#### Proof

Suppose that *N* is saturated w.r.t. $FInf$ and $Red ^{\mathcal {G}}$—i.e., $FInf (N) \subseteq Red _{\text {I}}^{\mathcal {G}}(N)$. We must show that ${\mathcal {G}}(N)$ is saturated w.r.t. $GInf$ and $Red$—i.e., $GInf ({\mathcal {G}}(N)) \subseteq Red _{\text {I}}({\mathcal {G}}(N))$.

Let $\iota ' \in GInf ({\mathcal {G}}(N))$. By assumption, $\iota '$ is contained in ${\mathcal {G}}( FInf (N))$ or $Red _{\text {I}}({\mathcal {G}}(N))$. In the second case, we are done immediately. In the first case, $\iota ' \in {\mathcal {G}}(\iota )$ for some $\iota \in FInf (N) \subseteq Red _{\text {I}}^{\mathcal {G}}(N)$ with ${\mathcal {G}}(\iota ) \not = undef$, so by definition of $Red _{\text {I}}^{\mathcal {G}}$ we have again $\iota ' \in Red _{\text {I}}({\mathcal {G}}(N))$. $\square$

An inference in $GInf ({\mathcal {G}}(N))$ is called *liftable* if it is contained in ${\mathcal {G}}( FInf (N))$. Using this terminology, we can rephrase the lemma as follows: If *N* is saturated and every unliftable inference from ${\mathcal {G}}(N)$ is redundant w.r.t. ${\mathcal {G}}(N)$, then ${\mathcal {G}}(N)$ is saturated.

#### Theorem 32

If $( GInf , Red )$ is statically refutationally complete w.r.t. $\models$, and if we have $GInf ({\mathcal {G}}(N)) \subseteq {\mathcal {G}}( FInf (N)) \cup Red _{\text {I}}({\mathcal {G}}(N))$ for every $N \subseteq {\mathbf {F}}$ that is saturated w.r.t. $FInf$ and $Red ^{\mathcal {G}}$, then $( FInf , Red ^{\mathcal {G}})$ is statically refutationally complete w.r.t. $\models _{\mathcal {G}}$.

#### Proof

Assume $( GInf , Red )$ is statically refutationally complete w.r.t. $\models$. Assume $N \subseteq {\mathbf {F}}$ is saturated w.r.t. $FInf$ and $Red ^{\mathcal {G}}$ and assume that $N \models _{\mathcal {G}}\bot$ for some $\bot \in {\mathbf {F}}_\bot$. We must show that $\bot ' \in N$ for some $\bot ' \in {\mathbf {F}}_\bot$.

By definition of $\models _{\mathcal {G}}$, we know that ${\mathcal {G}}(N) \models {\mathcal {G}}(\bot )$. By property (G1) of grounding functions, ${\mathcal {G}}(\bot )$ is a nonempty subset of ${\mathbf {G}}_\bot$. Let $\bot _{\mathbf {G}}\in {\mathcal {G}}(\bot )$. Then ${\mathcal {G}}(N) \models {\mathcal {G}}(\bot ) \models \{\bot _{\mathbf {G}}\}$.

By the previous lemma, we know that ${\mathcal {G}}(N)$ is saturated w.r.t. $GInf$ and $Red$, so there exists some $\bot '_{\mathbf {G}}\in {\mathbf {G}}_\bot$ such that $\bot '_{\mathbf {G}}\in {\mathcal {G}}(N)$. Hence $\bot '_{\mathbf {G}}\in {\mathcal {G}}(C)$ for some $C \in N$, which implies $C \in {\mathbf {F}}_\bot$ by property (G2) of grounding functions. Now define $\bot ' := C$. $\square$

#### Example 33

In ordered binary resolution without selection [6, 35], all inferences are liftable, as demonstrated below. Let $\Sigma$ be a first-order signature containing at least one constant, let ${\mathbf {F}}$ be the set of all $\Sigma$-clauses without equality, and let ${\mathbf {G}}$ be the set of all ground $\Sigma$-clauses without equality. Let $FInf$ and $GInf$ be the sets of all resolution or factoring inferences from clauses in respectively ${\mathbf {F}}$ and ${\mathbf {G}}$ that satisfy the given ordering restrictions, and let ${\mathcal {G}}$ be the function that maps every clause $C \in {\mathbf {F}}$ to the set of all its ground instances $C\theta$ and that maps every inference $(C_n,\ldots ,C_0) \in FInf$ to the set of all $(C_n\theta ,\ldots ,C_0\theta ) \in GInf$. Then every resolution inference in $GInf$ from ground instances of clauses in *N* has the form 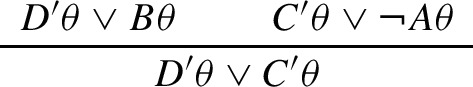 with $A\theta = B\theta$ and is contained in ${\mathcal {G}}(\iota )$ for some inference $\iota \in FInf (N)$ of the form 
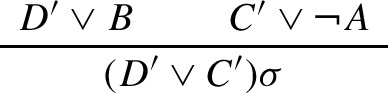
 with $\sigma = {{\,\mathrm{mgu}\,}}(A,B)$, and analogously for factoring inferences.

Therefore, the static refutational completeness of $GInf$ implies the static refutational completeness of $FInf$.

The liftability result above holds also for ordered binary resolution with selection, provided that the selection function $fsel$ on ${\mathbf {F}}$ and the selection function $gsel$ on ${\mathbf {G}}$ are such that every clause $D \in {\mathcal {G}}(N)$ inherits the selection of at least one clause $C \in N$ for which $D \in {\mathcal {G}}(C)$. One can show that for every $N \subseteq {\mathbf {G}}$ and $fsel$, such a $gsel$ exists. However, this $gsel$ depends on *N*, and therefore Theorem 32 is not applicable. We will discuss this issue further in Sect. 3.3.

#### Example 34

In the superposition calculus without selection [5], all inferences are liftable, except superpositions at or below a variable position. Let $\Sigma$ be a first-order signature containing at least one constant and no predicate symbols except $\approx$, let ${\mathbf {F}}$ be the set of all $\Sigma$-clauses with equality, and let ${\mathbf {G}}$ be the set of all ground $\Sigma$-clauses with equality. Let $FInf$ and $GInf$ be the sets of all superposition, equality resolution, and equality factoring inferences from clauses in respectively ${\mathbf {F}}$ and ${\mathbf {G}}$ that satisfy the given ordering restrictions, and let ${\mathcal {G}}$ be the function that maps every clause $C \in {\mathbf {F}}$ to the set of all its ground instances $C\theta$ and that maps every inference $(C_n,\ldots ,C_0) \in FInf$ to the set of all $(C_n\theta ,\ldots ,C_0\theta ) \in GInf$. Then every equality resolution or equality factoring inference from ground instances of clauses in *N* is contained in ${\mathcal {G}}(\iota )$ for some inference $\iota \in FInf (N)$. The same applies to superposition inferences 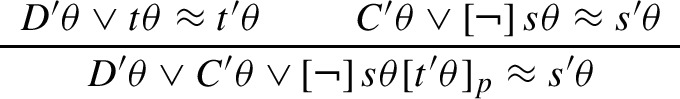


with $s\theta |_p = t\theta$, provided that *p* is a position of *s* and $s|_p$ is not a variable. Otherwise, $p = p_1 p_2$ for some variable *x* occurring in *s* at the position $p_1$, so $x\theta |_{p_2} = t\theta$. In this case, define $\theta '$ such that $x\theta ' = x\theta [t'\theta ]_{p_2}$ and $y\theta ' = y\theta$ for $y \not = x$. By congruence, the conclusion of the inference is entailed by the first premise (which is necessarily smaller than the second) and $C'\theta ' \vee [\lnot ]\, s\theta ' \approx s'\theta '$. The ordering restrictions of the calculus require that $t\theta \succ t'\theta$; hence the latter clause is also smaller than the second premise. By the usual redundancy criterion for superposition, this renders the inference redundant w.r.t. *N*.

Like for ordered resolution, the static refutational completeness of $GInf$ implies the static refutational completeness of $FInf$.

### Adding Tiebreaker Orderings

We now strengthen the ${\mathcal {G}}$-lifting of redundancy criteria introduced in the previous subsection to also support subsumption deletion. Let ${\sqsupset } = (\sqsupset _D)_{D \in {\mathbf {G}}}$ be a ${\mathbf {G}}$-indexed family of strict partial orderings on ${\mathbf {F}}$ that are well founded (i.e., for every *D*, $\sqsupset _D$ there exists no infinite descending chain $C_0 \sqsupset _D C_1 \sqsupset _D \cdots$). We define $Red _{\text {F}}^{{\mathcal {G}},\sqsupset } : \mathcal {P}({\mathbf {F}}) \rightarrow \mathcal {P}({\mathbf {F}})$ as follows:Notice how ${\sqsupset _D}$ is used to break ties between *C* and $C'$, possibly making *C* redundant. We call $Red ^{{\mathcal {G}},\sqsupset } := ( Red _{\text {I}}^{\mathcal {G}}, Red _{\text {F}}^{{\mathcal {G}},\sqsupset })$ the $({\mathcal {G}},\sqsupset )$-*lifting* of $Red$.

For nearly all applications (with a notable exception in Example 49 below), the orderings $\sqsupset _D$ agree for all $D \in {\mathbf {G}}$. In these cases, we may take $\sqsupset$ as a single well-founded strict partial ordering, rather than as a ${\mathbf {G}}$-indexed family of such orderings. We get the previously defined $Red ^{\mathcal {G}}= ( Red _{\text {I}}^{\mathcal {G}}, Red _{\text {F}}^{\mathcal {G}})$ as a special case of $Red ^{{\mathcal {G}},\sqsupset } = ( Red _{\text {I}}^{\mathcal {G}}, Red _{\text {F}}^{{\mathcal {G}},\sqsupset })$ by setting ${\sqsupset _D} := \emptyset$—i.e., the empty strict partial ordering on ${\mathbf {F}}$—for every $D \in {\mathbf {G}}$.

As demonstrated by the following lemma, we may assume without loss of generality that the formula $C'$ in the definition of $Red _{\text {F}}^{{\mathcal {G}},\sqsupset }$ is contained in $N \setminus Red _{\text {F}}^{{\mathcal {G}},\sqsupset }(N)$:

#### Lemma 35

$C \in Red _{\text {F}}^{{\mathcal {G}},\sqsupset }(N)$ if and only if for every $D \in {\mathcal {G}}(C)$ we have $D \in Red _{\text {F}}({\mathcal {G}}(N))$ or there exists $C' \in N \setminus Red _{\text {F}}^{{\mathcal {G}},\sqsupset }(N)$ such that $C \sqsupset _DC'$ and $D \in {\mathcal {G}}(C')$.

#### Proof

The “if” direction is trivial. For the “only if” direction, assume that $C \in Red _{\text {F}}^{{\mathcal {G}},\sqsupset }(N)$ and $D \in {\mathcal {G}}(C)$. By definition, $D \in Red _{\text {F}}({\mathcal {G}}(N))$ or there exists $C' \in N$ such that $C \sqsupset _DC'$ and $D \in {\mathcal {G}}(C')$. If $D \in Red _{\text {F}}({\mathcal {G}}(N))$, we are done. Let $D \notin Red _{\text {F}}({\mathcal {G}}(N))$. By well-foundedness of $\sqsupset _D$, there exists a minimal formula $C' \in N$ w.r.t. $\sqsupset _D$ such that $C \sqsupset _DC'$ and $D \in {\mathcal {G}}(C')$. Assume that $C'$ were contained in $Red _{\text {F}}^{{\mathcal {G}},\sqsupset }(N)$. Since $D \notin Red _{\text {F}}({\mathcal {G}}(N))$, there exists $C'' \in N$ such that $C' \sqsupset _DC''$ and $D \in {\mathcal {G}}(C'')$. But then $C \sqsupset _DC''$, contradicting the minimality of $C'$. So $C' \in N \setminus Red _{\text {F}}^{{\mathcal {G}},\sqsupset }(N)$. $\square$

Next, we show that $( Red _{\text {I}}^{\mathcal {G}}, Red _{\text {F}}^{{\mathcal {G}},\sqsupset })$ is a redundancy criterion. We start with a technical lemma:

#### Lemma 36

$${\mathcal {G}}(N) \setminus Red _{\text {F}}({\mathcal {G}}(N)) \subseteq {\mathcal {G}}(N \setminus Red _{\text {F}}^{{\mathcal {G}},\sqsupset }(N))$$.

#### Proof

Let $D \in {\mathcal {G}}(N) \setminus Red _{\text {F}}({\mathcal {G}}(N))$. Since $D \in {\mathcal {G}}(N)$, there exists $C \in N$ with $D \in {\mathcal {G}}(C)$. Let *C* be a minimal formula with this property w.r.t. $\sqsupset _D$.

Assume that $C \in Red _{\text {F}}^{{\mathcal {G}},\sqsupset }(N)$. Then, by definition, $D \in Red _{\text {F}}({\mathcal {G}}(N))$ or there exists $C' \in N$ such that $C \sqsupset _DC'$ and $D \in {\mathcal {G}}(C')$. The first property contradicts our initial assumption, whereas the second property contradicts the minimality of *C*. So $C \notin Red _{\text {F}}^{{\mathcal {G}},\sqsupset }(N)$ and thus $D \in {\mathcal {G}}(N \setminus Red _{\text {F}}^{{\mathcal {G}},\sqsupset }(N))$. $\square$

We can now show that $( Red _{\text {I}}^{\mathcal {G}}, Red _{\text {F}}^{{\mathcal {G}},\sqsupset })$ satisfies the properties (R1)–(R4) of redundancy criteria:

#### Lemma 37

If $N \models _{\mathcal {G}}\{\bot \}$ for some $\bot \in {\mathbf {F}}_\bot$, then $N \setminus Red _{\text {F}}^{{\mathcal {G}},\sqsupset }(N) \models _{\mathcal {G}}\{\bot \}$.

#### Proof

Let $\bot \in {\mathbf {F}}_\bot$ and suppose that $N \models _{\mathcal {G}}\{\bot \}$—i.e., ${\mathcal {G}}(N) \models {\mathcal {G}}(\{\bot \})$. Since ${\mathcal {G}}(\{\bot \})$ contains some $\bot ' \in {\mathbf {G}}_\bot$ by property (G1) of grounding functions, we have ${\mathcal {G}}(N) \models {\mathcal {G}}(\{\bot \}) \models \{\bot '\}$. By property (R1) of redundancy criteria, this implies ${\mathcal {G}}(N) \setminus Red _{\text {F}}({\mathcal {G}}(N)) \models \{\bot '\}$. Furthermore, by Lemma 36, ${\mathcal {G}}(N) \setminus Red _{\text {F}}({\mathcal {G}}(N)) \subseteq {\mathcal {G}}(N \setminus Red _{\text {F}}^{{\mathcal {G}},\sqsupset }(N))$, and therefore ${\mathcal {G}}(N \setminus Red _{\text {F}}^{{\mathcal {G}},\sqsupset }(N)) \models {\mathcal {G}}(N) \setminus Red _{\text {F}}({\mathcal {G}}(N))$. Combining the two relations, we obtain ${\mathcal {G}}(N \setminus Red _{\text {F}}^{{\mathcal {G}},\sqsupset }(N)) \models \{\bot '\} \models {\mathcal {G}}(\{\bot \})$. By definition of $\models _{\mathcal {G}}$ and property (G1) of grounding functions, this means $N \setminus Red _{\text {F}}^{{\mathcal {G}},\sqsupset }(N) \models _{\mathcal {G}}\{\bot \}$, as required. $\square$

#### Lemma 38

If $N \subseteq N'$, then $Red _{\text {F}}^{{\mathcal {G}},\sqsupset }(N) \subseteq Red _{\text {F}}^{{\mathcal {G}},\sqsupset }(N')$ and $Red _{\text {I}}^{\mathcal {G}}(N) \subseteq Red _{\text {I}}^{\mathcal {G}}(N')$.

#### Proof

Obvious. $$\square $$

#### Lemma 39

If $$N' \subseteq Red _{\text {F}}^{{\mathcal {G}},\sqsupset }(N)$$, then $$ Red _{\text {F}}^{{\mathcal {G}},\sqsupset }(N) \subseteq Red _{\text {F}}^{{\mathcal {G}},\sqsupset }(N \setminus N')$$.

#### Proof

Let $N' \subseteq Red _{\text {F}}^{{\mathcal {G}},\sqsupset }(N)$, let $C \in Red _{\text {F}}^{{\mathcal {G}},\sqsupset }(N)$. Then, by Lemma 35, for every $D \in {\mathcal {G}}(C)$ we have $D \in Red _{\text {F}}({\mathcal {G}}(N))$ or there exists $C' \in N \setminus Red _{\text {F}}^{{\mathcal {G}},\sqsupset }(N)$ such that $C \sqsupset _DC'$ and $D \in {\mathcal {G}}(C')$.

Case 1: $D \in Red _{\text {F}}({\mathcal {G}}(N))$. By property (R3), $D \in Red _{\text {F}}({\mathcal {G}}(N) \setminus Red _{\text {F}}({\mathcal {G}}(N)))$. Since ${\mathcal {G}}(N) \setminus Red _{\text {F}}({\mathcal {G}}(N)) \subseteq {\mathcal {G}}(N \setminus Red _{\text {F}}^{{\mathcal {G}},\sqsupset }(N)) \subseteq {\mathcal {G}}(N \setminus N')$, this implies $D \in Red _{\text {F}}({\mathcal {G}}(N \setminus N'))$.

Case 2: $D \notin Red _{\text {F}}({\mathcal {G}}(N))$ and there exists $C' \in N \setminus Red _{\text {F}}^{{\mathcal {G}},\sqsupset }(N)$ such that $C \sqsupset _DC'$ and $D \in {\mathcal {G}}(C')$. Since $N \setminus Red _{\text {F}}^{{\mathcal {G}},\sqsupset }(N) \subseteq N \setminus N'$, we get $C' \in N \setminus N'$.

Since every $D \in {\mathcal {G}}(C)$ is either contained in $Red _{\text {F}}({\mathcal {G}}(N \setminus N'))$ or in ${\mathcal {G}}(C')$ for some $C' \in N \setminus N'$ with $C \sqsupset _DC'$, we conclude that $C \in Red _{\text {F}}^{{\mathcal {G}},\sqsupset }(N \setminus N')$. $\square$

#### Lemma 40

If $$N' \subseteq Red _{\text {F}}^{{\mathcal {G}},\sqsupset }(N)$$, then $$ Red _{\text {I}}^{\mathcal {G}}(N) \subseteq Red _{\text {I}}^{\mathcal {G}}(N \setminus N')$$.

#### Proof

Let $$N' \subseteq Red _{\text {F}}^{{\mathcal {G}},\sqsupset }(N)$$, let $$\iota \in Red _{\text {I}}^{\mathcal {G}}(N)$$.

If ${\mathcal {G}}(\iota ) \not = undef$, then every $\iota ' \in {\mathcal {G}}(\iota )$ is contained in $Red _{\text {I}}({\mathcal {G}}(N))$, and by property (R3) also in $Red _{\text {I}}({\mathcal {G}}(N) \setminus Red _{\text {F}}({\mathcal {G}}(N)))$. Furthermore, since ${\mathcal {G}}(N) \setminus Red _{\text {F}}({\mathcal {G}}(N)) \subseteq {\mathcal {G}}(N \setminus Red _{\text {F}}^{{\mathcal {G}},\sqsupset }(N)) \subseteq {\mathcal {G}}(N \setminus N')$ by Lemma 36, this implies $\iota ' \in Red _{\text {I}}({\mathcal {G}}(N \setminus N'))$ by (R2). Since every $\iota ' \in {\mathcal {G}}(\iota )$ is contained in $Red _{\text {I}}({\mathcal {G}}(N \setminus N'))$, we conclude that $\iota \in Red _{\text {I}}^{\mathcal {G}}(N \setminus N')$.

Otherwise ${\mathcal {G}}(\iota ) = undef$. Then ${\mathcal {G}}( concl (\iota )) \subseteq {\mathcal {G}}(N) \cup Red _{\text {F}}({\mathcal {G}}(N)) = ({\mathcal {G}}(N) \setminus Red _{\text {F}}({\mathcal {G}}(N))) \cup Red _{\text {F}}({\mathcal {G}}(N))$. Let $D \in {\mathcal {G}}( concl (\iota ))$. We consider two cases: If $D \in {\mathcal {G}}(N) \setminus Red _{\text {F}}({\mathcal {G}}(N))$, then by Lemma 36, $D \in {\mathcal {G}}(N \setminus Red _{\text {F}}^{{\mathcal {G}},\sqsupset }(N)) \subseteq {\mathcal {G}}(N \setminus N')$. Otherwise $D \in Red _{\text {F}}({\mathcal {G}}(N))$, then by (R3) $D \in Red _{\text {F}}({\mathcal {G}}(N) \setminus Red _{\text {F}}({\mathcal {G}}(N)))$. Since ${\mathcal {G}}(N) \setminus Red _{\text {F}}({\mathcal {G}}(N)) \subseteq {\mathcal {G}}(N \setminus Red _{\text {F}}^{{\mathcal {G}},\sqsupset }(N)) \subseteq {\mathcal {G}}(N \setminus N')$, this implies $D \in Red _{\text {F}}({\mathcal {G}}(N \setminus N'))$. Combining both cases, we obtain ${\mathcal {G}}( concl (\iota )) \in {\mathcal {G}}(N \setminus N') \cup Red _{\text {F}}({\mathcal {G}}(N \setminus N'))$, hence $\iota \in Red _{\text {I}}^{\mathcal {G}}(N \setminus N')$. $\square$

#### Lemma 41

If $\iota \in FInf$ and $concl (\iota ) \in N$, then $\iota \in Red _{\text {I}}^{\mathcal {G}}(N)$.

#### Proof

Let $\iota \in FInf$ such that $concl (\iota ) \in N$. If ${\mathcal {G}}(\iota ) \not = undef$, then by property (G3) of grounding functions, ${\mathcal {G}}(\iota )$ is a subset of $Red _{\text {I}}({\mathcal {G}}( concl (\iota )))$, which in turn is a subset of $Red _{\text {I}}({\mathcal {G}}(N))$. So $\iota \in Red _{\text {I}}^{\mathcal {G}}(N)$.

Otherwise, ${\mathcal {G}}(\iota ) = undef$. Then $concl (\iota ) \in N$ implies ${\mathcal {G}}( concl (\iota )) \subseteq {\mathcal {G}}(N)$, so again $\iota \in Red _{\text {I}}^{\mathcal {G}}(N)$.$\square$

By combining Lemmas 37– 41, we obtain our first main result, generalizing Theorem 30:

#### Theorem 42

Let $Red$ be a redundancy criterion for $\models$ and $GInf$, let ${\mathcal {G}}$ be a grounding function from ${\mathbf {F}}$ and $FInf$ to ${\mathbf {G}}$ and $GInf$, and let ${\sqsupset } = (\sqsupset _D)_{D \in {\mathbf {G}}}$ be a ${\mathbf {G}}$-indexed family of well-founded strict partial orderings on ${\mathbf {F}}$. Then the $({\mathcal {G}},\sqsupset )$-lifting $Red ^{{\mathcal {G}},\sqsupset }$ of $Red$ is a redundancy criterion for $\models _{\mathcal {G}}$ and $FInf$.

Observe that $\sqsupset$ appears only in the second component of $Red ^{{\mathcal {G}},\sqsupset } = ( Red _{\text {I}}^{\mathcal {G}}, Red _{\text {F}}^{{\mathcal {G}},\sqsupset })$ and that the definitions of a saturated set and of static refutational completeness do not depend on the second component of a redundancy criterion. The following lemmas are immediate consequences of these observations:

#### Lemma 43

A set $N \subseteq {\mathbf {F}}$ is saturated w.r.t. $FInf$ and $Red ^{{\mathcal {G}},\sqsupset }$ if and only if it is saturated w.r.t. $FInf$ and $Red ^{{\mathcal {G}},\emptyset }$.

#### Lemma 44

$( FInf , Red ^{{\mathcal {G}},\sqsupset })$ is statically refutationally complete w.r.t. $\models _{\mathcal {G}}$ if and only if $( FInf , Red ^{{\mathcal {G}},\emptyset })$ is statically refutationally complete w.r.t. $\models _{\mathcal {G}}$.

Combining Lemmas 10 and 44, we obtain our second main result:

#### Theorem 45

Let $Red$ be a redundancy criterion for $\models$ and $GInf$, let ${\mathcal {G}}$ be a grounding function from ${\mathbf {F}}$ and $FInf$ to ${\mathbf {G}}$ and $GInf$, and let ${\sqsupset } = (\sqsupset _D)_{D \in {\mathbf {G}}}$ be a ${\mathbf {G}}$-indexed family of well-founded strict partial orderings on ${\mathbf {F}}$. If $( FInf , Red ^{{\mathcal {G}},\emptyset })$ is statically refutationally complete w.r.t. $\models _{\mathcal {G}}$, then $( FInf , Red ^{{\mathcal {G}},\sqsupset })$ is dynamically refutationally complete w.r.t. $\models _{\mathcal {G}}$.

#### Example 46

For resolution or superposition in standard first-order logic, we can define the *instantiation* quasi-ordering  on clauses by  if and only if $C = C'\sigma$ for some substitution $\sigma$. In particular, if *C* and $C'$ are $\alpha$-renamings of each other, then  and .

The instantiation ordering  is well founded. By choosing , we obtain a criterion $Red ^{{\mathcal {G}},\sqsupset }$ that includes standard redundancy (Example 3) and also supports subsumption deletion. (It is customary to define subsumption so that *C* is subsumed by $C'$ if $C = C'\sigma \vee D$ for some substitution $\sigma$ and some possibly empty clause *D*, but since the case where *D* is nonempty is already supported by the standard redundancy criterion, the instantiation ordering  is sufficient.)

Similarly, for proof calculi modulo commutativity (C) or associativity and commutativity (AC), we can let  be true if there exists a substitution $\sigma$ such that *C* equals $C'\sigma$ up to the equational theory (C or AC). The relation  is then again well founded.

#### Example 47

For higher-order calculi such as higher-order resolution [25] and $\lambda$-superposition [14], the instantiation ordering is not well founded, as witnessed by the chain

#### Example 48

In constraint superposition with ordering constraints [29], a ground instance of a constrained clause $C\,[\![K]\!]$ is defined as a ground clause $C\theta$ for which the constraint $K\theta$ evaluates to true. One can then define the quasi-ordering  by stating that  if and only if every ground instance of $C\,[\![K]\!]$ is a ground instance of $C'\,[\![K']\!]$. Again, the ordering . is not well founded, sinceis an infinite chain if $\succ$ is a simplification ordering.

Even if the instantiation ordering for some logic is not well founded, as in the two examples above, we can always define $\sqsupset$ as the intersection of the instantiation quasi-ordering and an appropriate ordering based on formula sizes or weights, such as 
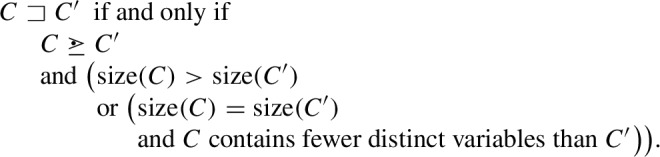
 Conversely, the $\sqsupset$ relation can be more general than subsumption. In Sect. 4, we will use it to justify the movement of formulas between sets in the given clause procedure.

#### Example 49

There are a few applications, notably for superposition-based decision procedures [7], where one would like to define  using the reverse instantiation ordering .

In this way, a clause ${\mathsf {P}}(x)$ would for example become redundant in the presence of the clauses ${\mathsf {P}}({\mathsf {b}})$ and ${\mathsf {P}}({\mathsf {c}})$, provided that ${\mathsf {b}}$ and ${\mathsf {c}}$ are the only two ground terms. The reverse instantiation ordering is not well founded on the set of all first-order clauses: . However, it is well founded if we restrict it to the set of generalizations  of a fixed ground clause *D*, so that we may in fact define . For this application, the possibility of taking $\sqsupset$ to be a ${\mathbf {G}}$-indexed family of well-founded strict partial orderings, as opposed to a single such ordering, is vital.

### Intersections of Liftings

The results of the previous subsection can be extended in a straightforward way to intersections of lifted redundancy criteria. As before, let $${\mathbf {F}}$$ and $${\mathbf {G}}$$ be two sets of formulas, and let $$ FInf $$ be an $${\mathbf {F}}$$-inference system. In addition, let *Q* be a nonempty set. For every $$q \in Q$$, let $${\models ^q}$$ be a consequence relation over $${\mathbf {G}}$$, let $$ GInf ^q$$ be a $${\mathbf {G}}$$-inference system, let $$ Red ^q$$ be a redundancy criterion for $${\models ^q}$$ and $$ GInf ^q$$, and let $${\mathcal {G}}^q$$ be a grounding function from $${\mathbf {F}}$$ and $$ FInf $$ to $${\mathbf {G}}$$ and $$ GInf ^q$$. Let $${\sqsupset } := (\sqsupset _D)_{D \in {\mathbf {G}}}$$ be a $${\mathbf {G}}$$-indexed family of well-founded strict partial orderings on $${\mathbf {F}}$$.^3^

For each $$q \in Q$$, we know by Theorem 42 that the $${({\mathcal {G}}^q,\emptyset )}$$-lifting $$ Red ^{q,{\mathcal {G}}^q,\emptyset } = ( Red _{\text {I}}^{q,{\mathcal {G}}^q}, Red _{\text {F}}^{q,{\mathcal {G}}^q,\emptyset })$$ and the $${({\mathcal {G}}^q,\sqsupset )}$$-lifting $$ Red ^{q,{\mathcal {G}}^q,\sqsupset } = ( Red _{\text {I}}^{q,{\mathcal {G}}^q}, Red _{\text {F}}^{q,{\mathcal {G}}^q,\sqsupset })$$ of $$ Red ^q$$ are redundancy criteria for $$\models ^{q}_{{\mathcal {G}}^q}$$ and $$ FInf $$. Consequently, by Lemma 24 the intersections are redundancy criteria for $${\models }^\cap _{\mathcal {G}}:= \bigcap _{q \in Q} {\models ^{q}_{{\mathcal {G}}^q}}$$ and $$ FInf $$.

We get the following analogue of Theorem 32.

#### Theorem 50

If $( GInf ^q, Red ^q)$ is statically refutationally complete w.r.t. $\models ^q$ for every $q \in Q$, and if for every $N \subseteq {\mathbf {F}}$ that is saturated w.r.t. $FInf$ and $Red ^{\cap {\mathcal {G}}}$ there exists a *q* such that $GInf ^q({\mathcal {G}}^q(N)) \subseteq {\mathcal {G}}^q( FInf (N)) \cup Red _{\text {I}}^q({\mathcal {G}}^q(N))$, then $( FInf , Red ^{\cap {\mathcal {G}}})$ is statically refutationally complete w.r.t. ${\models }^\cap _{\mathcal {G}}$.

#### Proof

Assume that $( GInf ^q, Red ^q)$ is statically refutationally complete w.r.t. $\models ^q$ for every $q \in Q$ and that for every $N \subseteq {\mathbf {F}}$ that is saturated w.r.t. $FInf$ and $Red ^{\cap {\mathcal {G}}}$ there exists a *q* such that $GInf ^q({\mathcal {G}}^q(N)) \subseteq {\mathcal {G}}^q( FInf (N)) \cup Red _{\text {I}}^q({\mathcal {G}}^q(N))$.

Let $$N \subseteq {\mathbf {F}}$$ be saturated w.r.t. $$ FInf $$ and $$ Red ^{\cap {\mathcal {G}}}$$ and assume that $$N \models ^\cap _{\mathcal {G}}\{\bot \}$$ for some $$\bot \in {\mathbf {F}}_\bot $$. We must show that $$\bot ' \in N$$ for some $$\bot ' \in {\mathbf {F}}_\bot $$. First, we know that there exists a *q* such that $$ GInf ^q({\mathcal {G}}^q(N)) \subseteq {\mathcal {G}}^q( FInf (N)) \cup Red _{\text {I}}^q({\mathcal {G}}^q(N))$$. Since $$ Red ^{\cap {\mathcal {G}}} = \bigcap _{q \in Q} Red ^{q,{\mathcal {G}}^q,\emptyset }$$, we know by Lemma 25 that *N* is saturated w.r.t. $$ FInf $$ and the $${({\mathcal {G}}^q,\emptyset )}$$-lifting $$ Red ^{q,{\mathcal {G}}^q,\emptyset }$$ of $$ Red ^q$$. Therefore, by Lemma 31, $${\mathcal {G}}^q(N)$$ is saturated w.r.t. $$ GInf $$ and $$ Red ^q$$.

Furthermore, $N \models ^\cap _{\mathcal {G}}\{\bot \}$ implies $N \models ^{q}_{{\mathcal {G}}^q} \{\bot \}$, and since $\models ^{q}_{{\mathcal {G}}^q}$ is the ${\mathcal {G}}^q$-lifting of $\models ^q$, this is equivalent to ${\mathcal {G}}^q(N) \models ^q {\mathcal {G}}^q(\bot )$. By property (G1) of grounding functions, ${\mathcal {G}}^q(\bot )$ is a nonempty subset of ${\mathbf {G}}_\bot$. Let $\bot _{\mathbf {G}}\in {\mathcal {G}}^q(\bot )$. Then ${\mathcal {G}}^q(N) \models {\mathcal {G}}^q(\bot ) \models \{\bot _{\mathbf {G}}\}$.

Since $${\mathcal {G}}^q(N)$$ is saturated w.r.t. $$ GInf $$ and $$ Red ^q$$, there must exist some $$\bot '_{\mathbf {G}}\in {\mathbf {G}}_\bot $$ such that $$\bot '_{\mathbf {G}}\in {\mathcal {G}}^q(N)$$. Hence $$\bot '_{\mathbf {G}}\in {\mathcal {G}}^q(C)$$ for some $$C \in N$$, which implies $$C \in {\mathbf {F}}_\bot $$ by property (G2) of grounding functions. Now define $$\bot ' := C$$. $$\square $$

Since the first components of $Red ^{\cap {\mathcal {G}}}$ and $Red ^{\cap {\mathcal {G}},\sqsupset }$ agree, we obtain the analogues of Lemmas 43 and 44 and Theorem 45:

#### Lemma 51

A set $N \subseteq {\mathbf {F}}$ is saturated w.r.t. $FInf$ and $Red ^{\cap {\mathcal {G}},\sqsupset }$ if and only if it is saturated w.r.t. $FInf$ and $Red ^{\cap {\mathcal {G}}}$.

#### Lemma 52

$( FInf , Red ^{\cap {\mathcal {G}},\sqsupset })$ is statically refutationally complete w.r.t. $\models ^\cap _{\mathcal {G}}$ if and only if $( FInf , Red ^{\cap {\mathcal {G}}})$ is statically refutationally complete w.r.t. $\models ^\cap _{\mathcal {G}}$.

#### Theorem 53

If $( FInf , Red ^{\cap {\mathcal {G}}})$ is statically refutationally complete w.r.t. $\models ^\cap _{\mathcal {G}}$, then $( FInf , Red ^{\cap {\mathcal {G}},\sqsupset })$ is dynamically refutationally complete w.r.t. $\models ^\cap _{\mathcal {G}}$.

#### Example 54

Intersections of liftings are needed to support selection functions in ordered resolution [6] and superposition [5]. The calculus $FInf$ is parameterized by a function $fsel$ on the set ${\mathbf {F}}$ of first-order clauses that selects a subset of the negative literals in each $C \in {\mathbf {F}}$. There are several ways to extend $fsel$ to a selection function $gsel$ on the set ${\mathbf {G}}$ of ground clauses such that for every $D \in {\mathbf {G}}$ there exists some $C \in {\mathbf {F}}$ such that $D = C\theta$ and *D* and *C* have corresponding selected literals.

For example, if $$ fsel $$ selects the first literal in $$C_1 \,=\, \lnot {\mathsf {P}}(x) \vee \lnot {\mathsf {Q}}({\mathsf {c}})$$ and the second literal in $$C_2 \,=\, \lnot {\mathsf {P}}({\mathsf {b}}) \vee \lnot {\mathsf {Q}}(y)$$, then $$ gsel $$ could select the first literal in $$D \,=\, \lnot {\mathsf {P}}({\mathsf {b}}) \vee \lnot {\mathsf {Q}}({\mathsf {c}})$$ (as in $$C_1$$) or the second literal (as in $$C_2$$). For every such $$ gsel $$, $$\models ^{ gsel }$$ is first-order entailment, $$ GInf ^{ gsel }$$ is the set of ground inferences satisfying $${ gsel }$$, and $$ Red ^{ gsel }$$ is the redundancy criterion for $$ GInf ^{ gsel }$$. The grounding function $${\mathcal {G}}^{ gsel }$$ maps $$C \in {\mathbf {F}}$$ to $$\{D \in {\mathbf {G}} \mid D = C\theta \text { for some }\theta \}$$ and $$\iota \in FInf $$ to the set of ground instances of $$\iota $$ in $$ GInf ^{ gsel }$$ with corresponding literals selected in the premises.

If $\iota$ is the $FInf$-inference 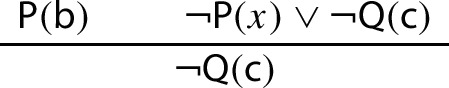 where the first literal in the second premise $C_1$ is selected by $fsel$, then 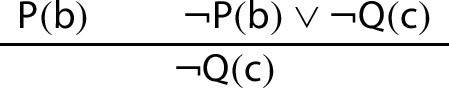 is contained in $${\mathcal {G}}^{ gsel }(\iota ) \subseteq GInf ^{ gsel }$$ if $$ gsel $$ selects the first literal in the right premise (as in $$C_1$$) but it is not contained in $$ GInf ^{ gsel }$$ (and hence not in $${\mathcal {G}}^{ gsel }(\iota )$$) if $$ gsel $$ selects the second literal in the right premise (as in $$C_2$$).

In the static refutational completeness proof, only one $gsel$ is needed, but this $gsel$ depends on the limit of a derivation and is not known *during* the derivation. Therefore, fairness must be guaranteed w.r.t. $Red _{\text {I}}^{{ gsel },{\mathcal {G}}^{ gsel }}$ for every possible extension $gsel$ of $fsel$. Checking $Red _{\text {I}}^{\cap {\mathcal {G}}}$ amounts to a worst-case analysis, where we must assume that every ground instance $C\theta \in {\mathbf {G}}$ of a premise $C \in {\mathbf {F}}$ inherits the selection of *C*.

#### Example 55

Intersections of liftings are also necessary for constraint superposition calculi (Nieuwenhuis and Rubio [29]). Here the calculus $$ FInf $$ operates on the set $${\mathbf {F}}$$ of first-order clauses with (ordering and equality) constraints. For a convergent rewrite system *R*, $$\models ^R$$ is first-order entailment up to *R* on the set $${\mathbf {G}}$$ of unconstrained ground clauses, $$ GInf ^R$$ is the set of ground superposition inferences, and $$ Red ^R$$ is redundancy up to *R*. The grounding function $${\mathcal {G}}^R$$ maps $$C\,[\![K]\!] \in {\mathbf {F}}$$ to $$\{D \in {\mathbf {G}} \mid D = C\theta , K\theta = \text {true}, x\theta \text { is} $$R$$\text {-irreducible for all } x \}$$ (except in degenerate cases where *x* occurs only in positive literals $$x \approx t$$) and $$\iota \in FInf $$ to the set of ground instances of $$\iota $$ where the premises and conclusion of $${\mathcal {G}}^R(\iota )$$ are the $${\mathcal {G}}^R$$-ground instances of the premises and conclusion of $$\iota $$. In the static refutational completeness proof, only one particular *R* is needed, but this *R* is not known during a derivation, so fairness must be guaranteed w.r.t. $$ Red _{\text {I}}^{R,{\mathcal {G}}^R}$$ for every convergent rewrite system *R*.

To obtain a practically useful criterion, the intersection $Red ^{\cap {\mathcal {G}}}$ must be approximated appropriately; compare Nieuwenhuis and Rubio’s Definition 6.5 (which corresponds to our $Red ^{\cap {\mathcal {G}}}$) to their Lemma 6.18.

#### Example 56

Some calculi have inference rules that introduce Skolem function symbols. An example is the $\delta$-elimination rule of Ganzinger and Stuber [23]. At the nonground level, the difficulty is that whenever we generate a conclusion with a fresh symbol ${\mathsf {sk}}_i$, we need to mark all other instances of the rule with ${\mathsf {sk}}_j$ ($j \not = i$) as redundant; otherwise, we would end up generating lots of needless conclusions. This determinism can be avoided by encoding enough information to identify the rule instance in the subscript *i*.

At the ground level, a second difficulty arises. The ground inference cannot simply introduce a nullary Skolem symbol—in general, this would not match the behavior of the corresponding nonground inference. Instead, it must guess both the Skolem symbol and its argument list. This guessing can be achieved using a selection function that takes the rule instance as argument and returns the Skolem term. We can then lift the ground inference by taking the intersection of all possible selection functions.

Almost every redundancy criterion for a nonground inference system $FInf$ that can be found in the literature can be written as $Red ^{{\mathcal {G}},\emptyset }$ for some grounding function ${\mathcal {G}}$ from ${\mathbf {F}}$ and $FInf$ to ${\mathbf {G}}$ and $GInf$, and some redundancy criterion $Red$ for $GInf$, or as an intersection $Red ^{\cap {\mathcal {G}}}$ of such criteria. As Theorem 53 demonstrates, every static refutational completeness result for $FInf$ and $Red ^{\cap {\mathcal {G}}}$—which does not generally support the deletion of subsumed formulas during a run—immediately yields a dynamic refutational completeness result for $FInf$ and $Red ^{\cap {\mathcal {G}},\sqsupset }$—which permits the deletion of subsumed formulas during a run, provided that they are larger according to $\sqsupset$.

### Adding Labels

In practice, the orderings $\sqsupset _D$ used in $({\mathcal {G}},\sqsupset )$-liftings often depend on meta-information about a formula, such as its age or the way in which it has been processed so far during a derivation. To capture this meta-information, we extend formulas and inference systems in a rather trivial way with labels.

As before, let ${\mathbf {F}}$ and ${\mathbf {G}}$ be two sets of formulas, let $FInf$ be an ${\mathbf {F}}$-inference system, let $GInf$ be a ${\mathbf {G}}$-inference system, let ${\models } \subseteq \mathcal {P}({\mathbf {G}}) \times \mathcal {P}({\mathbf {G}})$ be a consequence relation over ${\mathbf {G}}$, let $Red$ be a redundancy criterion for $\models$ and $GInf$, and let ${\mathcal {G}}$ be a grounding function from ${\mathbf {F}}$ and $FInf$ to ${\mathbf {G}}$ and $GInf$.

Let ${\mathbf {L}}$ be a nonempty set of *labels*. Define ${\mathbf {FL}}:= {\mathbf {F}}\times {\mathbf {L}}$ and ${\mathbf {FL}}_\bot := {\mathbf {F}}_\bot \times {\mathbf {L}}$. Notice that there are at least as many false values in ${\mathbf {FL}}$ as there are labels in ${\mathbf {L}}$. We use $\mathcal {M}, \mathcal {N}$ to denote labeled formula sets. Given a set $\mathcal {N}\subseteq {\mathbf {FL}}$, let $\lfloor \mathcal {N}\rfloor := \{C \mid (C,l) \in \mathcal {N} \}$ denote the set of formulas without their labels.

We call an ${\mathbf {FL}}$-inference system $FLInf$ a *labeled version* of $FInf$ if it has the following properties:

1. for every inference $(C_n,\dots ,C_0) \in FInf$ and every tuple $(l_1,\dots ,l_n) \in {\mathbf {L}}^n$, there exists an $l_0 \in {\mathbf {L}}$ and an inference $((C_n,l_n),\dots ,(C_0,l_0)) \in FLInf$;
2. if $\iota = ((C_n,l_n),\dots ,(C_0,l_0))$ is an inference in $FLInf$, then $(C_n,\dots ,C_0)$ is an inference in $FInf$, denoted by $\lfloor \iota \rfloor$.

In other words, whenever there is an $FInf$-inference from some premises, there is a corresponding $FLInf$-inference from the labeled premises (regardless of the labeling), and whenever there is an $FLInf$-inference from labeled premises, there is a corresponding $FInf$-inference from the unlabeled premises.

Let $FLInf$ be a labeled version of $FInf$. Define ${\mathcal {G}}_{\mathbf {L}}$ by ${\mathcal {G}}_{\mathbf {L}}(C,l) := {\mathcal {G}}(C)$ for every $(C,l) \in {\mathbf {FL}}$ and by ${\mathcal {G}}_{\mathbf {L}}(\iota ) := {\mathcal {G}}(\lfloor \iota \rfloor )$ for every $\iota \in FLInf$. The following lemmas are then obvious:

#### Lemma 57

${\mathcal {G}}_{\mathbf {L}}$ is a grounding function from ${\mathbf {FL}}$ and $FLInf$ to ${\mathbf {G}}$ and $GInf$.

Let $\models _{{\mathcal {G}}_{\mathbf {L}}}$ be the ${\mathcal {G}}_{\mathbf {L}}$-lifting of $\models$. Let $Red ^{{{\mathcal {G}}_{\mathbf {L}}},\emptyset }$ be the $({\mathcal {G}}_{\mathbf {L}},\emptyset )$-lifting of $Red$.

#### Lemma 58

$\mathcal {N}\models _{{\mathcal {G}}_{\mathbf {L}}}\mathcal {N}'$ if and only if $\lfloor \mathcal {N}\rfloor \models _{\mathcal {G}}\lfloor \mathcal {N}'\rfloor$.

#### Lemma 59

If a set $\mathcal {N}\subseteq {\mathbf {FL}}$ is saturated w.r.t. $FLInf$ and $Red ^{{{\mathcal {G}}_{\mathbf {L}}},\emptyset }$, then $\lfloor \mathcal {N}\rfloor \subseteq {\mathbf {F}}$ is saturated w.r.t. $FInf$ and $Red ^{{\mathcal {G}},\emptyset }$.

#### Lemma 60

If $( FInf , Red ^{{\mathcal {G}},\emptyset })$ is statically refutationally complete w.r.t. $\models _{\mathcal {G}}$, then $( FLInf , Red ^{{{\mathcal {G}}_{\mathbf {L}}},\emptyset })$ is statically refutationally complete w.r.t. $\models _{{\mathcal {G}}_{\mathbf {L}}}$.

The extension to intersections of redundancy criteria is also straightforward. Let $${\mathbf {F}}$$ and $${\mathbf {G}}$$ be two sets of formulas, and let $$ FInf $$ be an $${\mathbf {F}}$$-inference system. Let *Q* be a nonempty set. For every $$q \in Q$$, let $${\models ^q}$$ be a consequence relation over $${\mathbf {G}}$$, let $$ GInf ^q$$ be a $${\mathbf {G}}$$-inference system, let $$ Red ^q$$ be a redundancy criterion for $${\models ^q}$$ and $$ GInf ^q$$, and let $${\mathcal {G}}^q$$ be a grounding function from $${\mathbf {F}}$$ and $$ FInf $$ to $${\mathbf {G}}$$ and $$ GInf ^q$$. Then for every $$q \in Q$$, the $${({\mathcal {G}}^q,\emptyset )}$$-lifting $$ Red ^{q,{\mathcal {G}}^q,\emptyset }$$ of $$ Red ^q$$ is a redundancy criterion for the $${\mathcal {G}}^q$$-lifting $$\models ^{q}_{{\mathcal {G}}^q}$$ of $$\models ^q$$ and $$ FInf $$, and so $$ Red ^{\cap {\mathcal {G}}}$$ is a redundancy criterion for $${\models }^\cap _{\mathcal {G}}$$ and $$ FInf $$.

Now let $${\mathbf {L}}$$ be a nonempty set of labels, and define $${\mathbf {FL}}$$, $${\mathbf {FL}}_\bot $$, and $$ FLInf $$ as above. For every $$q \in Q$$, define the function $${\mathcal {G}}_{\mathbf {L}}^q$$ by $${\mathcal {G}}_{\mathbf {L}}^q(C,l) := {\mathcal {G}}^q(C)$$ for every $$(C,l) \in {\mathbf {FL}}$$ and by $${\mathcal {G}}_{\mathbf {L}}^q(\iota ) := {\mathcal {G}}^q(\lfloor \iota \rfloor )$$ for every $$\iota \in FLInf $$. By Lemma 57, every $${\mathcal {G}}_{\mathbf {L}}^q$$ is a grounding function from $${\mathbf {FL}}$$ and $$ FLInf $$ to $${\mathbf {G}}$$ and $$ GInf ^q$$. Then for every $$q \in Q$$, the $${({\mathcal {G}}_{\mathbf {L}}^q,\emptyset )}$$-lifting $$ Red ^{q,{\mathcal {G}}_{\mathbf {L}}^q} = ( Red _{\text {I}}^{q,{\mathcal {G}}_{\mathbf {L}}^q}, Red _{\text {F}}^{q,{\mathcal {G}}_{\mathbf {L}}^q,\emptyset })$$ of $$ Red ^q$$ is a redundancy criterion for the $${\mathcal {G}}_{\mathbf {L}}^q$$-lifting $$\models _{{\mathcal {G}}_{\mathbf {L}}^q}^q$$ of $$\models ^q$$ and $$ FLInf $$, and so

$$\begin{aligned} Red ^{\cap {\mathcal {G}}_{\mathbf {L}}} := ( Red _{\text {I}}^{\cap {\mathcal {G}}_{\mathbf {L}}}, Red _{\text {F}}^{\cap {\mathcal {G}}_{\mathbf {L}}}) := \Bigl ({\bigcap _{q \in Q} Red _{\text {I}}^{q,{\mathcal {G}}_{\mathbf {L}}^q},\; \bigcap _{q \in Q} Red _{\text {F}}^{q,{\mathcal {G}}_{\mathbf {L}}^q,\emptyset }}\Bigr ) \end{aligned}$$

is a redundancy criterion for ${\models }^\cap _{{\mathcal {G}}_{\mathbf {L}}} := \bigcap _{q \in Q} {\models _{{\mathcal {G}}_{\mathbf {L}}^q}^q}$ and $FLInf$.

Analogously to Lemmas 58–60, we obtain the following results:

#### Lemma 61

$\mathcal {N}\models ^\cap _{{\mathcal {G}}_{\mathbf {L}}} \mathcal {N}'$ if and only if $\lfloor \mathcal {N}\rfloor \models ^\cap _{\mathcal {G}}\lfloor \mathcal {N}'\rfloor$.

#### Lemma 62

If a set $\mathcal {N}\subseteq {\mathbf {FL}}$ is saturated w.r.t. $FLInf$ and $Red ^{\cap {\mathcal {G}}_{\mathbf {L}}}$, then $\lfloor \mathcal {N}\rfloor \subseteq {\mathbf {F}}$ is saturated w.r.t. $FInf$ and $Red ^{\cap {\mathcal {G}}}$.

#### Theorem 63

If $( FInf , Red ^{\cap {\mathcal {G}}})$ is statically refutationally complete w.r.t. ${\models }^\cap _{\mathcal {G}}$, then $( FLInf , Red ^{\cap {\mathcal {G}}_{\mathbf {L}}})$ is statically refutationally complete w.r.t. $\models ^\cap _{{\mathcal {G}}_{\mathbf {L}}}$.

## Prover Architectures

We now use the above results to prove the refutational completeness of a popular prover architecture: the given clause procedure invented by McCune and Wos [28]. The architecture is parameterized by an inference system and a redundancy criterion. A generalization of the architecture decouples scheduling and computation of inferences, which has several benefits.

### Given Clause Procedure

For this section, we fix the following. Let $${\mathbf {F}}$$ and $${\mathbf {G}}$$ be two sets of formulas, and let $$ FInf $$ be an $${\mathbf {F}}$$-inference system without premise-free inferences. Let *Q* be a nonempty set. For every $$q \in Q$$, let $${\models ^q}$$ be a consequence relation over $${\mathbf {G}}$$, let $$ GInf ^q$$ be a $${\mathbf {G}}$$-inference system, let $$ Red ^q$$ be a redundancy criterion for $${\models ^q}$$ and $$ GInf ^q$$, and let $${\mathcal {G}}^q$$ be a grounding function from $${\mathbf {F}}$$ and $$ FInf $$ to $${\mathbf {G}}$$ and $$ GInf ^q$$. Assume $$( FInf , Red ^{\cap {\mathcal {G}}})$$ is statically refutationally complete w.r.t. $${\models }^\cap _{\mathcal {G}}$$.

Let ${\mathbf {L}}$ be a nonempty set of labels, let ${\mathbf {FL}}:= {\mathbf {F}}\times {\mathbf {L}}$, and let the ${\mathbf {FL}}$-inference system $FLInf$ be a labeled version of $FInf$. By Theorem 63, $( FLInf , Red ^{\cap {\mathcal {G}}_{\mathbf {L}}})$ is statically refutationally complete w.r.t. $\models ^\cap _{{\mathcal {G}}_{\mathbf {L}}}$.

Let  be an equivalence relation on ${\mathbf {F}}$, let  be a well-founded strict partial ordering on ${\mathbf {F}}$ such that  is compatible with  (i.e., , ,  implies ), such that  implies ${\mathcal {G}}^q(C) = {\mathcal {G}}^q(D)$ for all $q \in Q$, and such that  implies  for all $q \in Q$. We define . In practice,  is typically $\alpha$-renaming (or equality if formulas are considered up to $\alpha$-equivalence),  is either the instantiation ordering  (Example 46), provided it is well founded, or some well-founded ordering included in , and for every $q \in Q$, ${\mathcal {G}}^q$ maps every formula $C \in {\mathbf {F}}$ to the set of ground instances of *C*, possibly modulo some theory.

Let  be a well-founded strict partial ordering on ${\mathbf {L}}$. We define the ordering $\sqsupset$ on ${\mathbf {FL}}$ by $(C,l) \sqsupset (C',l')$ if either  or else  and . By Lemma 52, the static refutational completeness of $( FLInf , Red ^{\cap {\mathcal {G}}_{\mathbf {L}}})$ w.r.t. $\models ^\cap _{{\mathcal {G}}_{\mathbf {L}}}$ implies the static refutational completeness of $( FLInf , Red ^{\cap {\mathcal {G}}_{\mathbf {L}},\sqsupset })$, which by Lemma 10 implies the dynamic refutational completeness of $( FLInf , Red ^{\cap {\mathcal {G}}_{\mathbf {L}},\sqsupset })$.

This result may look intimidating, so let us unroll it. The ${\mathbf {FL}}$-inference system $FLInf$ is a labeled version of $FInf$, which means that we get an $FLInf$-inference by first omitting the labels of the premises $(C_n,l_n),\dots , (C_1,l_1)$, then performing an $FInf$-inference $(C_n,\dots ,C_0)$, and finally attaching an arbitrary label $l_0$ to the conclusion $C_0$. Since the labeled grounding functions ${\mathcal {G}}_{\mathbf {L}}^q$ differ from the corresponding unlabeled grounding functions ${\mathcal {G}}^q$ only by the omission of the labels and the first components of $Red ^{\cap {\mathcal {G}}_{\mathbf {L}},\sqsupset }$ and $Red ^{\cap {\mathcal {G}}_{\mathbf {L}}}$ agree, we get this result:

#### Lemma 64

An $FLInf$-inference $\iota$ is redundant w.r.t. $Red ^{\cap {\mathcal {G}}_{\mathbf {L}},\sqsupset }$ and $\mathcal {N}$ if and only if the underlying $FInf$-inference $\lfloor \iota \rfloor$ is redundant w.r.t. $Red ^{\cap {\mathcal {G}}}$ and $\lfloor \mathcal {N}\rfloor$.

For $Red _{\text {F}}^{\cap {\mathcal {G}}_{\mathbf {L}},\sqsupset }$, we can show that a labeled formula (*C*, *l*) is redundant if (i) *C* itself is redundant w.r.t. $Red _{\text {F}}^{\cap {\mathcal {G}}}$, or if (ii) *C* is -subsumed, or if (iii) *C* is a variant of another formula that occurs with a -smaller label. More formally:

#### Lemma 65

Let $\mathcal {N}\subseteq {\mathbf {FL}}$, and let (*C*, *l*) be a labeled formula. Then $(C,l) \in Red _{\text {F}}^{\cap {\mathcal {G}}_{\mathbf {L}},\sqsupset }(\mathcal {N})$ if one of the following conditions hold:

(i) $$C \in Red _{\text {F}}^{\cap {\mathcal {G}}}(\lfloor \mathcal {N}\rfloor )$$;
(ii) for some $C' \in \lfloor \mathcal {N}\rfloor$;
(iii) for some $(C',l') \in \mathcal {N}$ with .

#### Proof

(i) Let $C \in Red _{\text {F}}^{\cap {\mathcal {G}}}(\lfloor \mathcal {N}\rfloor )$. Then $C \in Red _{\text {F}}^{q,{\mathcal {G}}^q,\emptyset }(\lfloor \mathcal {N}\rfloor )$ for every $q \in Q$, which means that ${\mathcal {G}}^q(C) \subseteq Red _{\text {F}}^q({\mathcal {G}}^q(\lfloor \mathcal {N}\rfloor ))$. Now ${\mathcal {G}}_{\mathbf {L}}^q(C,l) = {\mathcal {G}}^q(C)$ and ${\mathcal {G}}^q(\lfloor \mathcal {N}\rfloor ) = {\mathcal {G}}_{\mathbf {L}}^q(\mathcal {N})$; hence ${\mathcal {G}}_{\mathbf {L}}^q(C,l) \subseteq Red _{\text {F}}^q({\mathcal {G}}_{\mathbf {L}}^q(\mathcal {N}))$, which implies $(C,l) \in Red _{\text {F}}^{q,{\mathcal {G}}_{\mathbf {L}}^q,\sqsupset }(\mathcal {N})$ for every $q \in Q$ and thus $(C,l) \in Red _{\text {F}}^{\cap {\mathcal {G}}_{\mathbf {L}},\sqsupset }(\mathcal {N})$.

(ii) Assume that  for some $C' \in \lfloor \mathcal {N}\rfloor$. Then there exists a label $l'$ such that $(C',l') \in \mathcal {N}$. By the definition of $\sqsupset$, we have $(C,l) \sqsupset (C',l')$. Furthermore, ${\mathcal {G}}^q(C) \subseteq {\mathcal {G}}^q(C')$ for all $q \in Q$. Therefore ${\mathcal {G}}_{\mathbf {L}}^q(C,l) = {\mathcal {G}}^q(C) \subseteq {\mathcal {G}}^q(C') = {\mathcal {G}}_{\mathbf {L}}^q(C',l')$, which implies $(C,l) \in Red _{\text {F}}^{q,{\mathcal {G}}_{\mathbf {L}}^q,\sqsupset }(\mathcal {N})$ for every $q \in Q$ and thus $(C,l) \in Red _{\text {F}}^{\cap {\mathcal {G}}_{\mathbf {L}},\sqsupset }(\mathcal {N})$.

(iii) If , the result follows from (ii). Otherwise  for some $(C',l') \in \mathcal {N}$ with . Then $(C,l) \sqsupset (C',l')$ and ${\mathcal {G}}^q(C) = {\mathcal {G}}^q(C')$, so ${\mathcal {G}}_{\mathbf {L}}^q(C,l) = {\mathcal {G}}^q(C) = {\mathcal {G}}^q(C') = {\mathcal {G}}_{\mathbf {L}}^q(C',l')$. This implies $(C,l) \in Red _{\text {F}}^{q,{\mathcal {G}}_{\mathbf {L}}^q,\sqsupset }(\mathcal {N})$ for every $q \in Q$; therefore, $(C,l) \in Red _{\text {F}}^{\cap {\mathcal {G}}_{\mathbf {L}},\sqsupset }(\mathcal {N})$. $\square$

The given clause procedure that lies at the heart of saturation provers can be presented and studied abstractly.^4^ We assume that the set of labels ${\mathbf {L}}$ contains at least two values, one of which is a distinguished -smallest value denoted by $\mathsf {active}$, and that the labeled version $FLInf$ of $FInf$ never assigns the label $\mathsf {active}$ to a conclusion.

The state of a prover is a set of labeled formulas. The label identifies to which formula set each formula belongs. The $\mathsf {active}$ label identifies the active formula set from the given clause procedure. The other, unspecified formula sets are considered passive. Given a set $\mathcal {N}$ and a label *l*, we define the projection $\mathcal {N}{\downarrow }_l$ as consisting only of the formulas labeled by *l*.

The given clause prover $\mathsf {GC}$ is defined as the following transition system:

Process 
  where $$\mathcal {M}\subseteq Red _{\text {F}}^{\cap {\mathcal {G}}_{\mathbf {L}},\sqsupset }(\mathcal {N}\cup \mathcal {M}')$$ and $$\mathcal {M}'{\downarrow }_\mathsf {active} = \emptyset $$
Infer 
  where $$l \not = \mathsf {active}$$, $$\mathcal {M}{\downarrow }_\mathsf {active} = \emptyset $$, and $$ FInf (\lfloor \mathcal {N}{\downarrow }_\mathsf {active}\rfloor , \{C\}) \subseteq Red _{\text {I}}^{\cap {\mathcal {G}}}(\lfloor \mathcal {N}\rfloor \cup \{C\} \cup \lfloor \mathcal {M}\rfloor )$$

The initial state consists of the input formulas, paired with arbitrary labels different from $\mathsf {active}$. A key invariant of the given clause procedure is that all inferences from active formulas are redundant w.r.t. the current set of formulas.

The Process rule covers most operations performed in a theorem prover. By Lemma 65, this includes

- deleting $Red _{\text {F}}^{\cap {\mathcal {G}}}$-redundant formulas with arbitrary labels and adding formulas that make other formulas $Red _{\text {F}}^{\cap {\mathcal {G}}}$-redundant (i.e., simplifying w.r.t. $Red _{\text {F}}^{\cap {\mathcal {G}}}$), by (i);
- deleting formulas that are -subsumed by other formulas with arbitrary labels, by (ii);
- deleting formulas that are -subsumed by other formulas with smaller labels, by (iii);
- replacing the label of a formula by a smaller label different from $\mathsf {active}$, also by (iii).

Like for $\rhd _ Red$, in practice the added formulas would normally be entailed by *N*, but we impose no soundness restrictions.

Infer is the only rule that puts a formula in the active set. It relabels a passive formula *C* to $\mathsf {active}$ and ensures that all inferences between *C* and the active formulas, including *C* itself, become redundant. Recall that by Lemma 64, $FLInf (\mathcal {N}{\downarrow }_\mathsf {active}, \{(C,\mathsf {active})\}) \subseteq Red _{\text {I}}^{\cap {\mathcal {G}}_{\mathbf {L}}}(\mathcal {N}\cup \{(C, \mathsf {active})\} \cup \mathcal {M})$ if and only if $FInf (\lfloor \mathcal {N}{\downarrow }_\mathsf {active}\rfloor , \{C\}) \subseteq Red _{\text {I}}^{\cap {\mathcal {G}}}(\lfloor \mathcal {N}\rfloor \cup \{C\} \cup \lfloor \mathcal {M}\rfloor )$. By property (R4) of redundancy criteria, every inference is redundant if its conclusion is contained in the set of formulas, and typically, inferences are in fact made redundant by adding their conclusions to any of the passive sets. Then, $\lfloor \mathcal {M}\rfloor$ equals $concl ( FInf (\lfloor \mathcal {N}{\downarrow }_\mathsf {active}\rfloor , \{C\}))$. There are, however, some techniques commonly implemented in theorem provers for which we need Infer’s side condition in full generality.

#### Lemma 66

Every -derivation is a $\rhd _{ Red ^{\cap {\mathcal {G}}_{\mathbf {L}},\sqsupset }}$-derivation.

#### Proof

We need to show that every labeled formula that is deleted in a -step is $Red ^{\cap {\mathcal {G}}_{\mathbf {L}},\sqsupset }$-redundant w.r.t. the remaining labeled formulas. For Process, this is trivial. For Infer, the only deleted formula is (*C*, *l*), which is $Red ^{\cap {\mathcal {G}}_{\mathbf {L}},\sqsupset }$-redundant w.r.t. $(C,{\mathsf {active}})$ by part (iii) of Lemma 65, since . $\square$

Since $( FLInf , Red ^{\cap {\mathcal {G}}_{\mathbf {L}},\sqsupset })$ is dynamically refutationally complete, it now suffices to show fairness to prove the refutational completeness of .

Given $k \in \mathbb {N} \cup \{\infty \}$, let $Inv _\mathcal {N}(k)$ denote the condition

$$\begin{aligned} \textstyle FLInf (\mathcal {N}{}_k{\downarrow }_\mathsf {active}) \subseteq \bigcup _{i=0}^k Red _{\text {I}}^{\cap {\mathcal {G}}_{\mathbf {L}}}(\mathcal {N}{}_i). \end{aligned}$$

If $(\mathcal {N}{}_i)_i$ is a $\rhd _{ Red ^{\cap {\mathcal {G}}_{\mathbf {L}},\sqsupset }}$-derivation and $k \in \mathbb {N}$, by (R2) and (R3), the right-hand side is equal to $Red _{\text {I}}^{\cap {\mathcal {G}}_{\mathbf {L}}}(\mathcal {N}{}_k)$. We will show that $Inv _\mathcal {N}(i)$ is an invariant of  and that it extends to the limit, enabling us to establish fairness: $FLInf (\mathcal {N}{}_\infty ) \subseteq \bigcup _i Red _{\text {I}}^{\cap {\mathcal {G}}_{\mathbf {L}}}(\mathcal {N}{}_i)$.

#### Lemma 67

Let $(\mathcal {N}{}_i)_i$ be a -derivation. If , then $Inv _\mathcal {N}(k)$ holds for all indices *k*.

#### Proof

Base case: The hypothesis $\mathcal {N}{}_0{\downarrow }_\mathsf {active} = \emptyset$ and the exclusion of premise-free inferences ensure that $FLInf ({\downarrow }_\mathsf {active}) = \emptyset$ and hence $Inv _\mathcal {N}(0)$ holds.

Case Process: Consider the step . We have the inclusion chain $FLInf (\mathcal {N}{}_{k+1} {\downarrow }_\mathsf {active}) \subseteq FLInf (\mathcal {N}{}_k {\downarrow }_\mathsf {active}) \subseteq \bigcup _{i=0}^k Red _{\text {I}}^{\cap {\mathcal {G}}_{\mathbf {L}}}(\mathcal {N}{}_i) \subseteq \bigcup _{i=0}^{k+1} Red _{\text {I}}^{\cap {\mathcal {G}}_{\mathbf {L}}}(\mathcal {N}{}_i)$. The first inclusion relies on Process’s side condition that $\mathcal {M}'{\downarrow }_\mathsf {active} = \emptyset$. The second inclusion corresponds to the induction hypothesis.

Case Infer: Consider the step . We assume $\iota \in FLInf (\mathcal {N}{}_{k+1} {\downarrow }_\mathsf {active})$ for some $\iota$ and show $\iota \in \bigcup _{i=0}^{k+1} Red _{\text {I}}^{\cap {\mathcal {G}}_{\mathbf {L}}}(\mathcal {N}{}_i)$. If $\iota \in FLInf (\mathcal {N}{\downarrow }_\mathsf {active}, (C, \mathsf {active}))$, then $\iota \in Red _{\text {I}}^{\cap {\mathcal {G}}_{\mathbf {L}}}(\mathcal {N}{}_{k+1})$ by Infer’s last side condition. Otherwise, $\iota \in FLInf (\mathcal {N}{\downarrow }_\mathsf {active})$ by Infer’s side condition that $\mathcal {M}{\downarrow }_\mathsf {active} = \emptyset$. By the induction hypothesis, $\iota \in \bigcup _{i=0}^k Red _{\text {I}}^{\cap {\mathcal {G}}_{\mathbf {L}}}(\mathcal {N}{}_i)$. In both cases, $\iota \in \bigcup _{i=0}^{k+1} Red _{\text {I}}^{\cap {\mathcal {G}}_{\mathbf {L}}}(\mathcal {N}{}_i)$. $\square$

#### Lemma 68

Let $(\mathcal {N}{}_i)_i$ be a nonempty $\mathcal {P}({\mathbf {FL}})$-sequence. If $Inv _\mathcal {N}(i)$ holds for all indices *i*, then $Inv _\mathcal {N}(\infty )$ holds.

#### Proof

We assume $\iota \in FLInf (\mathcal {N}{}_\infty {\downarrow }_\mathsf {active})$ for some $\iota$ and show $\iota \in \bigcup _{i} Red _{\text {I}}^{\cap {\mathcal {G}}_{\mathbf {L}}}(\mathcal {N}{}_i)$. For $\iota$ to be in $FLInf (\mathcal {N}{}_\infty {\downarrow }_\mathsf {active})$, all of its finitely many premises must be in $\mathcal {N}{}_\infty {\downarrow }_\mathsf {active}$. Therefore, there must exist an index *k* such that $\mathcal {N}{}_k {\downarrow }_\mathsf {active}$ contains all of them, and therefore $\iota \in FLInf (\mathcal {N}{}_k {\downarrow }_\mathsf {active})$. Since $Inv _\mathcal {N}(k)$ holds, $\iota \in \bigcup _{i=0}^k Red _{\text {I}}^{\cap {\mathcal {G}}_{\mathbf {L}}}(\mathcal {N}{}_i) \subseteq \bigcup _{i} Red _{\text {I}}^{\cap {\mathcal {G}}_{\mathbf {L}}}(\mathcal {N}{}_i)$. $\square$

#### Lemma 69

Let $(\mathcal {N}{}_i)_i$ be a -derivation. If  and $\mathcal {N}{}_\infty {\downarrow }_l = \emptyset$ for all $l \not = \mathrm {active}$, then $(\mathcal {N}{}_i)_i$ is fair.

#### Proof

By Lemmas 67 and 68, $FLInf (\mathcal {N}{}_\infty {\downarrow }_\text {active}) \subseteq \bigcup _i Red _{\text {I}}^{\cap {\mathcal {G}}_{\mathbf {L}}}(\mathcal {N}{}_i)$. By the second hypothesis, this inclusion simplifies to $FLInf (\mathcal {N}{}_\infty ) \subseteq \bigcup _i Red _{\text {I}}^{\cap {\mathcal {G}}_{\mathbf {L}}}(\mathcal {N}{}_i)$. $\square$

#### Theorem 70

Let $(\mathcal {N}{}_i)_i$ be a -derivation, where  and $\mathcal {N}{}_\infty {\downarrow }_l = \emptyset$ for all . If $\lfloor \mathcal {N}{}_0\rfloor \models ^\cap _{\mathcal {G}}\{\bot \}$ for some $\bot \in {\mathbf {F}}_\bot$, then some $\mathcal {N}{}_i$ contains $(\bot ',l)$ for some $\bot ' \in {\mathbf {F}}_\bot$ and $l \in {\mathbf {L}}$.

#### Proof

By Lemma 61, $\lfloor \mathcal {N}{}_0\rfloor \models ^\cap _{\mathcal {G}}\{\bot \}$ is equivalent to $\mathcal {N}{}_0 \models ^\cap _{{\mathcal {G}}_{\mathbf {L}}} \{(\bot ,\mathsf {active})\}$. By Lemmas 66 and 69, we know that $(\mathcal {N}{}_i)_i$ is a fair $\rhd _{ Red ^{\cap {\mathcal {G}}_{\mathbf {L}},\sqsupset }}$-derivation. Since $( FLInf , Red ^{\cap {\mathcal {G}}_{\mathbf {L}},\sqsupset })$ is dynamically refutationally complete, we can conclude that some $\mathcal {N}{}_i$ contains $(\bot ',l)$ for some $\bot ' \in {\mathbf {F}}_\bot$ and $l \in {\mathbf {L}}$. $\square$

#### Example 71

The following Otter loop [28, Sect. 2.3.1] prover $\mathsf {OL}$ is an instance of the given clause prover . This loop design is inspired by Weidenbach’s prover without splitting from his *Handbook* chapter [46, Tables 4–6]. The prover’s state is a five-tuple $N \mid X \mid P \mid Y \mid A$ of formula sets. The *N*, *P*, and *A* sets store the new, passive, and active formulas, respectively. The *X* and *Y* sets are subsingletons (i.e., sets of at most one element) that can store a chosen new or passive formula, respectively. Initial states are of the form $N \mid \emptyset \mid \emptyset \mid \emptyset \mid \emptyset$.

ChooseN: $$N \uplus \{C\} \mid \emptyset \mid P \mid \emptyset \mid A \,\Longrightarrow _\mathsf {OL}\, N \mid \{C\} \mid P \mid \emptyset \mid A$$
DeleteFwd: $$N \mid \{C\} \mid P \mid \emptyset \mid A \,\Longrightarrow _\mathsf {OL}\, N \mid \emptyset \mid P \mid \emptyset \mid A$$
  if $C \in Red _{\text {F}}^{\cap {\mathcal {G}}}(P \cup A)$ or  for some $C' \in P \cup A$
SimplifyFwd: $$N \mid \{C\} \mid P \mid \emptyset \mid A \,\Longrightarrow _\mathsf {OL}\, N \mid \{C'\} \mid P \mid \emptyset \mid A$$
  if $$C \in Red _{\text {F}}^{\cap {\mathcal {G}}}(P \cup A \cup \{C'\})$$
DeleteBwdP: $$N \mid \{C\} \mid P \uplus \{C'\} \mid \emptyset \mid A \,\Longrightarrow _\mathsf {OL}\, N \mid \{C\} \mid P \mid \emptyset \mid A$$
  if $$C' \in Red _{\text {F}}^{\cap {\mathcal {G}}}(\{C\})$$ or
SimplifyBwdP: $$N \mid \{C\} \mid P \uplus \{C'\} \mid \emptyset \mid A \,\Longrightarrow _\mathsf {OL}\, N \cup \{C''\} \mid \{C\} \mid P \mid \emptyset \mid A$$
  if $$C' \in Red _{\text {F}}^{\cap {\mathcal {G}}}(\{C,C''\})$$
DeleteBwdA: $$N \mid \{C\} \mid P \mid \emptyset \mid A \uplus \{C'\} \,\Longrightarrow _\mathsf {OL}\, N \mid \{C\} \mid P \mid \emptyset \mid A$$
  if $$C' \in Red _{\text {F}}^{\cap {\mathcal {G}}}(\{C\})$$ or
SimplifyBwdA: $$N \mid \{C\} \mid P \mid \emptyset \mid A \uplus \{C'\} \,\Longrightarrow _\mathsf {OL}\, N \cup \{C''\} \mid \{C\} \mid P \mid \emptyset \mid A$$
  if $$C' \in Red _{\text {F}}^{\cap {\mathcal {G}}}(\{C,C''\})$$
Transfer: $$N \mid \{C\} \mid P \mid \emptyset \mid A \,\Longrightarrow _\mathsf {OL}\, N \mid \emptyset \mid P \cup \{C\} \mid \emptyset \mid A$$
ChooseP: $$\emptyset \mid \emptyset \mid P \uplus \{C\} \mid \emptyset \mid A \,\Longrightarrow _\mathsf {OL}\, \emptyset \mid \emptyset \mid P \mid \{C\} \mid A$$
Infer: $$\emptyset \mid \emptyset \mid P \mid \{C\} \mid A \,\Longrightarrow _\mathsf {OL}\, M \mid \emptyset \mid P \mid \emptyset \mid A \cup \{C\}$$
  if $$ FInf (A, \{C\}) \subseteq Red _{\text {I}}^{\cap {\mathcal {G}}}(A \cup \{C\} \cup M)$$

Weidenbach identifies the *X* and *Y* components of $\mathsf {OL}$’s five-tuples; this is possible since the former is used only in his inner loop, whereas the latter is used only in his outer loop.

If we are interested in soundness, we can require that the formulas added by simplification and Infer are -entailed by the formulas in the state before the transition. This can be relaxed to consistency preservation—e.g., for calculi that perform skolemization.

A reasonable strategy for applying the $\mathsf {OL}$ rules is presented below. It relies on a well-founded ordering $\succ$ on formulas to ensure that the simplification rules actually “simplify” their target, preventing nontermination of the inner loop. It also assumes that $FInf (N, \{C\})$ is finite if *N* is finite.

1. Repeat while $N \cup P \not = \emptyset$ and $\bot \notin N \cup P \cup A$:
  1.1. Repeat while $$N \not = \emptyset $$:
    1.1.1. Apply ChooseN to retrieve the next formula *C* from the state’s *N* component, which is organized as a queue.
    1.1.2. Apply SimplifyFwd as long as the simplified formula $C'$ is $\succ$-smaller than the original formula *C*.
    1.1.3. If DeleteFwd is applicable, apply it.
    1.1.4. Otherwise:
      1.1.4.1. Apply DeleteBwdP and DeleteBwdA exhaustively.
      1.1.4.2. Apply SimplifyBwdP and SimplifyBwdA as long as the simplified formula $C''$ is $\succ$-smaller than the original formula $C'$.
      1.1.4.3. Apply Transfer.
  1.2 If $$P \not = \emptyset $$:
    1.2.1. Apply ChooseP. Make sure that the choice of *C* is fair.
    1.2.2. Apply Infer with $M = concl ( FInf (A, \{C\}))$.

Let $(N_i \mid X_i \mid P_i \mid Y_i \mid A_i)_i$ be a $\Longrightarrow _\mathsf {OL}$-derivation that follows the strategy, where $N_0$ is finite and $X_0 = P_0 = Y_0 = A_0 = \emptyset$. If the outer loop terminates because $\bot \in N \cup P \cup A$, the condition of dynamic refutational completeness is trivially satisfied. Otherwise, the argument is as follows. With each application of a rule other than Infer, the state, viewed as a multiset of labeled formulas, decreases w.r.t. the multiset extension of a relation that compares formulas using $\succ$ and breaks ties using  on the labels. This ensures no formula is left in *N* or *X* forever. The fair choice of *C* ensures that that no formula is left in *P* forever, and the application of Infer following ChooseP ensures the same about *Y*. As a result, we have $N_\infty = X_\infty = P_\infty = Y_\infty = \emptyset$. Therefore, by Theorem 70, $\mathsf {OL}$ is dynamically refutationally complete.

In most saturation calculi, $Red$ is defined in terms of some total well-founded ordering $\succ _{\mathbf {G}}$ on ${\mathbf {G}}$. We can then define $\succ$ so that $C \succ C'$ if the smallest element of ${\mathcal {G}}^q(C)$ is greater than the smallest element of ${\mathcal {G}}^q(C')$ w.r.t. $\succ _{\mathbf {G}}$, for some arbitrary fixed $q \in Q$. This allows a wide range of simplifications implemented in resolution or superposition provers.

To ensure fairness, the heuristic used to apply ChooseP must guarantee that no formula remains indefinitely in *P*. Fair choice strategies typically rely on formula age, which can be represented through labels. Consider labeled formulas (*C*, *t*), where *t* is the timestamp, and a labeled version of $\mathsf {OL}$ where formulas introduced by simplification or $\textsc {Infer}$ are labeled with strictly increasing timestamps.

#### Example 72

One fair formula choice strategy is to alternate between heuristically choosing *n* formulas and taking the formula with the smallest timestamp [28, Sect. 2.3.1].

#### Proof

By contradiction. Assume $P_\infty \not = \emptyset$. Consider the formula $(C, t) \in P_\infty$ with the smallest timestamp *t*. There exists an index *i* such that *C* is the formula with the smallest timestamp in $P_i$. After at most $n + 1$ applications of ChooseP, *C* will be chosen. $\square$

#### Example 73

Another fair option is to use an $\mathbb {N}$-valued weight function *w* that is strictly monotonic in the timestamp—i.e., for any unlabeled formula *C*, if $t < t'$, then $w(C,t) < w(C,t')$—and take a formula with the smallest weight [37, Sect. 4].

#### Proof

Consider the labeled formula (*C*, *t*) with the smallest weight in $P_\infty$. The weight function satisfies the inequation $n \le w(D,n)$ for every *n* and every unlabeled *D*. Therefore, after *w*(*C*, *t*) applications of Infer, new formulas introduced by simplification or Infer all have a weight larger than (*C*, *t*), and thus $\textsc {ChooseP}$ will eventually have to choose (*C*, *t*). $\square$

#### Example 74

In its superposition module [20], iProver implements a rule that eliminates the chosen passive clause, or *given clause*, if it is redundant w.r.t. a subset of its child clauses together with the active set. The following iProver loop prover $\mathsf {IL}$ captures this. It is based on  and consists of all the $\mathsf {OL}$ transition rules and of the following rule:

Replace : $$\emptyset \mid \emptyset \mid P \mid \{C\} \mid A \,\Longrightarrow _\mathsf {IL}\, M \mid \emptyset \mid P \mid \emptyset \mid A$$
  if either $C \in Red _{\text {F}}^{\cap {\mathcal {G}}}(A \cup M)$ or else $M = \{C'\}$ and

As *M*, iProver would use a set of possibly simplified clauses from $concl ( FInf (A, \{C\}))$.

#### Example 75

Bachmair and Ganzinger’s resolution prover $\mathsf {RP}$ [6, Sect. 4.3] is another instance of . It embodies both a concrete prover architecture and a concrete inference system: ordered resolution with selection ($\mathsf {O}^\succ _S$). States are triples $N \mid P \mid O$ of finite clause sets consisting of new, processed, and old (active) clauses, respectively. The instantiation relies on three labels . Subsumption can be supported as described in Example 46.

Tauto: $$N \cup \{C\} \mid P \mid O \,\Longrightarrow _\mathsf {RP}\, N \mid P \mid O$$
  if *C* is a tautology
DeleteFwd: $$N \cup \{C\} \mid P \mid O \,\Longrightarrow _\mathsf {RP}\, N \mid P \mid O$$
  if some clause in $P \cup O$ subsumes *C*
ReduceFwd: $$N \cup \{C \vee L\} \mid P \mid O \,\Longrightarrow _\mathsf {RP}\, N \cup \{C\} \mid P \mid O$$
  if there is a clause $D \vee L'$ in $P \cup O$ such that $\bar{L} = L'\sigma$ and $D\sigma \subseteq C$
DeleteBwdP: $$N \mid P \cup \{C\} \mid O \,\Longrightarrow _\mathsf {RP}\, N \mid P \mid O$$
  if some clause in *N* properly subsumes *C*
ReduceBwdP: $$N \mid P \cup \{C \vee L\} \mid O \,\Longrightarrow _\mathsf {RP}\, N \mid P \cup \{C\} \mid O$$
  if there is a clause $D \vee L'$ in *N* such that $\bar{L} = L'\sigma$ and $D\sigma \subseteq C$
DeleteBwdO: $$N \mid P \mid O \cup \{C\} \,\Longrightarrow _\mathsf {RP}\, N \mid P \mid O$$
  if some clause in *N* properly subsumes *C*
ReduceBwdO: $$N \mid P \mid O \cup \{C \vee L\} \,\Longrightarrow _\mathsf {RP}\, N \mid P \cup \{C\} \mid O$$
  if there is a clause $D \vee L'$ in *N* such that $\bar{L} = L'\sigma$ and $D\sigma \subseteq C$
Choose: $$N \cup \{C\} \mid P \mid O \,\Longrightarrow _\mathsf {RP}\, N \mid P \cup \{C\} \mid O$$
Infer: $$\emptyset \mid P \cup \{C\} \mid O \,\Longrightarrow _\mathsf {RP}\, N \mid P \mid O \cup \{C\}$$
  if $$N = concl (\mathsf {O}^\succ _S(O,C))$$

Let $(N_i \mid P_i \mid O_i)_i$ be a $\Longrightarrow _\mathsf {RP}$-derivation, where $P_0 = O_0 = \emptyset$. Since the transition system excluding Infer terminates [37, Sect. 4] and we can always apply Choose to empty *N*, we have $N_\infty = \emptyset$. The key restriction that is needed to ensure fairness is that the choice of *C* in Infer must be fair. This ensures $P_\infty = \emptyset$. Thus, by Theorem 70, $\mathsf {RP}$ is dynamically refutationally complete. Incidentally, our version of $\mathsf {RP}$ repairs a small mistake in Bachmair and Ganzinger’s definition of the notation $Inf (N,\{C\})$, used in the Infer rule [36, Sect. 7.1].

### Delayed Inferences

The given clause prover  presented in the previous subsection is sufficient to describe a prover based on an Otter loop as well as a basic DISCOUNT loop prover—which differs from the Otter loop prover $\mathsf {OL}$ in that the passive formulas are neither simplified or deleted using SimplifyBwdP or DeleteBwdP, nor are they used to simplify or delete other formulas in SimplifyFwd or DeleteFwd. To describe a DISCOUNT loop prover with orphan formula deletion, however, we need to extend .

An *orphan* formula is a passive formula generated by an inference for which at least one premise is no longer active.

To model orphan formula deletion, we need to decouple the scheduling of inferences and their computation. The same scheme can be used to model provers based on inference systems that contain premise-free inferences or that may generate infinitely many conclusions from finitely many premises. Yet another use of the scheme is to save memory: A delayed inference can be stored more compactly than a new formula, as a tuple of premises together with instructions on how to compute the conclusion.

The lazy given clause prover  generalizes . It is defined as the following transition system on pairs $(T,\mathcal {N})$, where *T* (“to do”) is a set of *scheduled* inferences and $\mathcal {N}$ is a set of labeled formulas. We use the same assumptions as for  except that we now permit premise-free inferences in $FInf$.

Process
  where $$\mathcal {M}\subseteq Red _{\text {F}}^{\cap {\mathcal {G}}_{\mathbf {L}},\sqsupset }(\mathcal {N}\cup \mathcal {M}')$$ and $$\mathcal {M}'{\downarrow }_\mathsf {active} = \emptyset $$
ScheduleInfer
  where $$l \not = \mathsf {active}$$ and $$T' = FInf (\lfloor \mathcal {N}{\downarrow }_\mathsf {active}\rfloor , \{C\})$$
ComputeInfer
  where $$\mathcal {M}{\downarrow }_\mathsf {active} = \emptyset $$ and $$\iota \in Red _{\text {I}}^{\cap {\mathcal {G}}}(\lfloor \mathcal {N}\cup \mathcal {M}\rfloor )$$
DeleteOrphans
  where $$T' \cap FInf (\lfloor \mathcal {N}{\downarrow }_\mathsf {active}\rfloor ) = \emptyset $$

Initial states are states $(T, \mathcal {N})$ such that *T* consists of all premise-free inferences of $FInf$ and $\mathcal {N}$ contains the input formulas paired with arbitrary labels different from $\mathsf {active}$. A key invariant of  is that all inferences from active formulas are either scheduled in *T* or redundant w.r.t. $\mathcal {N}$.

Process has the same behavior as the corresponding  rule, except for the additional *T* component, which it ignores.

The Infer rule of  is split into two parts in : ScheduleInfer relabels a passive formula *C* to $\mathsf {active}$ and puts all inferences between *C* and the active formulas, including *C* itself, into the set *T*. ComputeInfer removes an inference from *T* and ensures that it becomes redundant by adding appropriate labeled formulas to $\mathcal {N}$ (typically the conclusion of the inference).

DeleteOrphans can delete scheduled inferences from *T* if some of their premises have been deleted from $\mathcal {N}{\downarrow }_\mathsf {active}$ in the meantime by an application of Process. Note that the rule cannot delete premise-free inferences, since the side condition is then trivially false.

Abstractly, the *T* component of the state is a set of inferences $(C_n,\ldots ,C_0)$. In an actual implementation, it can be represented in different ways: as a set of compactly encoded recipes for computing the conclusion $C_0$ from the premises $(C_n,\ldots ,C_1)$ as in Waldmeister [24], or as a set of explicit formulas $C_0$ with information about their parents $(C_n,\ldots ,C_1)$ as in E [39]. In the latter case, some presimplifications may be performed on $C_0$; this could be modeled more faithfully by defining *T* as a set of pairs $(\iota , simp (C_0))$.

#### Lemma 76

If $(T_i,\mathcal {N}{}_i)_i$ is an -derivation, then $(\mathcal {N}{}_i)_i$ is a $\rhd _{ Red ^{\cap {\mathcal {G}}_{\mathbf {L}},\sqsupset }}$-derivation.

#### Proof

We must show that every labeled formula that is deleted in an -step from the $\mathcal {N}$ component is $Red ^{\cap {\mathcal {G}}_{\mathbf {L}},\sqsupset }$-redundant w.r.t. the remaining labeled formulas. For Process this is trivial. For ScheduleInfer, the only deleted formula is (*C*, *l*), which is $Red ^{\cap {\mathcal {G}}_{\mathbf {L}},\sqsupset }$-redundant w.r.t. $(C,\mathsf {active})$ by part (iii) of Lemma 65, since . Finally, the rules ComputeInfer and DeleteOrphans do not delete any formulas. $\square$

Given $k \in \mathbb {N} \cup \{\infty \}$, let $Inv _{T,\mathcal {N}}(k)$ denote the condition

$$\begin{aligned} \textstyle FLInf (\mathcal {N}{}_k{\downarrow }_\mathsf {active}) \subseteq \lceil T_k\rceil \cup \bigcup _{i=0}^k Red _{\text {I}}^{\cap {\mathcal {G}}_{\mathbf {L}}}(\mathcal {N}{}_i) \end{aligned}$$

where $\iota \in \lceil T\rceil$ if and only if $\lfloor \iota \rfloor \in T$ for every $\iota$ and every *T*. We will show that $Inv _{T,\mathcal {N}}(i)$ is an invariant of  and that it extends to the limit, enabling us to establish fairness.

#### Lemma 77

Let $(T_i, \mathcal {N}{}_i)_i$ be an -derivation. If  and $T_0 \supseteq FInf (\emptyset )$, then $Inv _{T,\mathcal {N}}(k)$ holds for all indices *k*.

#### Proof

Base case: The hypotheses ensure that .

Case Process: This case is essentially as in the proof of Lemma 67.

Case ScheduleInfer:Consider the step . We assume $\iota \in FLInf (\mathcal {N}{}_{k+1} {\downarrow }_\mathsf {active})$ for some $\iota$ and show $\iota \in \lceil T \cup T'\rceil \cup \bigcup _{i=0}^{k+1} Red _{\text {I}}^{\cap {\mathcal {G}}_{\mathbf {L}}}(\mathcal {N}{}_i)$. If $\iota \in FLInf ( \mathcal {N}{\downarrow }_\mathsf {active}, (C, \mathsf {active}))$, then $\lfloor \iota \rfloor \in T'$ by definition of $T'$ and thus $\iota \in \lceil T \cup T' \rceil$. Otherwise, $\iota \in FLInf (\mathcal {N}{\downarrow }_\mathsf {active})$. By the induction hypothesis, $\iota \in \lceil T \rceil \cup \bigcup _{i=0}^k Red _{\text {I}}^{\cap {\mathcal {G}}_{\mathbf {L}}}(\mathcal {N}{}_i)$. In both cases, $\iota \in \lceil T \cup T' \rceil \cup \bigcup _{i=0}^{k+1} Red _{\text {I}}^{\cap {\mathcal {G}}_{\mathbf {L}}}(\mathcal {N}{}_i)$.

Case ComputeInfer:Consider the step . We assume $\iota \in FLInf (\mathcal {N}{}_{k+1} {\downarrow }_\mathsf {active})$ for some $\iota$ and show $\iota \in \lceil T \rceil \cup \bigcup _{i=0}^{k+1} Red _{\text {I}}^{\cap {\mathcal {G}}_{\mathbf {L}}}(\mathcal {N}{}_i)$. By ComputeInfer’s side condition that $\mathcal {M}{\downarrow }_\mathsf {active} = \emptyset$, we have that $\iota \in FLInf (\mathcal {N}{\downarrow }_\mathsf {active})$. By the induction hypothesis, $\iota \in \lceil T \cup \{\iota '\}\rceil \cup \bigcup _{i=0}^k Red _{\text {I}}^{\cap {\mathcal {G}}_{\mathbf {L}}}(\mathcal {N}{}_i)$. If $\iota \in \lceil \{\iota '\} \rceil$, then $\iota \in Red _{\text {I}}^{\cap {\mathcal {G}}_{\mathbf {L}}}(\mathcal {N}\cup \mathcal {M})$ by ComputeInfer’s last side condition. Otherwise, $\iota \in \lceil T \rceil \cup \bigcup _{i=0}^k Red _{\text {I}}^{\cap {\mathcal {G}}_{\mathbf {L}}}(\mathcal {N}{}_i)$. In both cases, $\iota \in \lceil T \rceil \cup \bigcup _{i=0}^{k+1} Red _{\text {I}}^{\cap {\mathcal {G}}_{\mathbf {L}}}(\mathcal {N}{}_i)$.

Case DeleteOrphans: Consider the transition . We assume $\iota \in FLInf (\mathcal {N}{\downarrow }_\mathsf {active})$ for some $\iota$. By the induction hypothesis, $\iota \in \lceil T \cup T'\rceil \cup \bigcup _{i=0}^{k+1} Red _{\text {I}}^{\cap {\mathcal {G}}_{\mathbf {L}}}(\mathcal {N}{}_i)$. Since $\iota \notin \lceil T'\rceil$ by DeleteOrphans’s side condition, we have $\iota \in \lceil T\rceil \cup \bigcup _{i=0}^{k+1} Red _{\text {I}}^{\cap {\mathcal {G}}_{\mathbf {L}}}(\mathcal {N}{}_i)$. $\square$

#### Lemma 78

Let $(T_i, \mathcal {N}{}_i)_i$ be a nonempty $(\mathcal {P}( FInf )\times \mathcal {P}({\mathbf {FL}}))$-sequence. If $Inv _{T,\mathcal {N}}(i)$ holds for all indices *i*, then $Inv _{T,\mathcal {N}}(\infty )$ holds.

#### Proof

We assume $\iota \in FLInf (\mathcal {N}{}_\infty {\downarrow }_\mathsf {active})$ for some $\iota$ and show $\iota \in \lceil T_\infty \rceil \cup \bigcup _{i} Red _{\text {I}}^{\cap {\mathcal {G}}_{\mathbf {L}}}(\mathcal {N}{}_i)$. Assume $\iota \notin \lceil T_\infty \rceil$. Clearly, there must exist an index *k* such that $\mathcal {N}{}_k {\downarrow }_\mathsf {active}$ contains all of $\iota$’s premises and $\iota \notin \lceil T_k \rceil$. Therefore $\iota \in FLInf (\mathcal {N}{}_k {\downarrow }_\mathsf {active})$. Since $Inv _{T,\mathcal {N}}(k)$ holds, $\iota \in \bigcup _{i=0}^k Red _{\text {I}}^{\cap {\mathcal {G}}_{\mathbf {L}}}(\mathcal {N}{}_i) \subseteq \bigcup _{i} Red _{\text {I}}^{\cap {\mathcal {G}}_{\mathbf {L}}}(\mathcal {N}{}_i)$. $\square$

#### Lemma 79

Let $(T_i,\mathcal {N}{}_i)_i$ be an -derivation. If , $\mathcal {N}{}_\infty {\downarrow }_l = \emptyset$ for all , $T_0 \supseteq FInf (\emptyset )$, and $T_\infty = \emptyset$, then $(\mathcal {N}{}_i)_i$ is fair.

#### Proof

By Lemmas 67 and 68, . By the second and fourth hypotheses, this inclusion simplifies to $FLInf (\mathcal {N}{}_\infty ) \subseteq \bigcup _i Red _{\text {I}}^{\cap {\mathcal {G}}_{\mathbf {L}}}(\mathcal {N}{}_i)$. $\square$

#### Theorem 80

Let $(T_i,\mathcal {N}{}_i)_i$ be an -derivation, where , $\mathcal {N}{}_\infty {\downarrow }_l = \emptyset$ for all , and $T_\infty = \emptyset$. If $\lfloor \mathcal {N}{}_0\rfloor \models ^\cap _{\mathcal {G}}\{\bot \}$ for some $\bot \in {\mathbf {F}}_\bot$, then some $\mathcal {N}{}_i$ contains $(\bot ',l)$ for some $\bot ' \in {\mathbf {F}}_\bot$ and $l \in {\mathbf {L}}$.

#### Proof

By Lemma 61, $\lfloor \mathcal {N}{}_0\rfloor \models ^\cap _{\mathcal {G}}\{\bot \}$ is equivalent to $\mathcal {N}{}_0 \models ^\cap _{{\mathcal {G}}_{\mathbf {L}}} \{(\bot ,\mathsf {active})\}$. By Lemmas 76 and 79, we know that $(\mathcal {N}{}_i)_i$ is a fair $\rhd _{ Red ^{\cap {\mathcal {G}}_{\mathbf {L}},\sqsupset }}$-derivation. Since $( FLInf , Red ^{\cap {\mathcal {G}}_{\mathbf {L}},\sqsupset })$ is dynamically refutationally complete, we can conclude that some $\mathcal {N}{}_i$ contains $(\bot ',l)$ for some $\bot ' \in {\mathbf {F}}_\bot$ and $l \in {\mathbf {L}}$. $\square$

#### Example 81

The following DISCOUNT loop [1] prover $\mathsf {DL}$ is an instance of the lazy given clause prover . This loop design is inspired by Schulz’s description of E [39] but omits E’s presimplification of $concl (\iota )$. The prover’s state is a four-tuple $T \mid P \mid Y \mid A$, where *T* is a set of inferences and *P*, *Y*, *A* are sets of formulas. The *T*, *P*, and *A* sets correspond to the scheduled inferences, the passive formulas, and the active formulas, respectively. The *Y* set is a subsingleton that can store a chosen passive formula. Initial states have the form $T \mid P \mid \emptyset \mid \emptyset$, where *T* is the set of all premise-free inferences of $FInf$.

ComputeInfer: $$T \uplus \{\iota \} \mid P \mid \emptyset \mid A \,\Longrightarrow _\mathsf {DL}\, T \mid P \mid \{C\} \mid A$$
  if $$\iota \in Red _{\text {I}}^{\cap {\mathcal {G}}}(A \cup \{C\})$$
ChooseP: $$T \mid P \uplus \{C\} \mid \emptyset \mid A \,\Longrightarrow _\mathsf {DL}\, T \mid P \mid \{C\} \mid A$$
DeleteFwd: $$T \mid P \mid \{C\} \mid A \,\Longrightarrow _\mathsf {DL}\, T \mid P \mid \emptyset \mid A$$
  if $C \in Red _{\text {F}}^{\cap {\mathcal {G}}}(A)$ or  for some $C' \in A$
SimplifyFwd: $$T \mid P \mid \{C\} \mid A \,\Longrightarrow _\mathsf {DL}\, T \mid P \mid \{C'\} \mid A$$
  if $$C \in Red _{\text {F}}^{\cap {\mathcal {G}}}(A \cup \{C'\})$$
DeleteBwd: $$T \mid P \mid \{C\} \mid A \uplus \{C'\} \,\Longrightarrow _\mathsf {DL}\, T \mid P \mid \{C\} \mid A$$
  if $$C' \in Red _{\text {F}}^{\cap {\mathcal {G}}}(\{C\})$$ or
SimplifyBwd: $$T \mid P \mid \{C\} \mid A \uplus \{C'\} \,\Longrightarrow _\mathsf {DL}\, T \mid P \cup \{C''\} \mid \{C\} \mid A$$
  if $$C' \in Red _{\text {F}}^{\cap {\mathcal {G}}}(\{C,C''\})$$
ScheduleInfer: $$T \mid P \mid \{C\} \mid A \,\Longrightarrow _\mathsf {DL}\, T \cup T' \mid P \mid \emptyset \mid A \cup \{C\}$$
  if $$T' = FInf (A, \{C\})$$
DeleteOrphans: $$T \uplus T' \mid P \mid Y \mid A \,\Longrightarrow _\mathsf {DL}\, T \mid P \mid Y \mid A$$
  if $$T' \cap FInf (A) = \emptyset $$

A reasonable strategy for applying the $\mathsf {DL}$ rules is presented below. It relies on a well-founded ordering $\succ$ on formulas to make sure that the simplification rules actually simplify their target in some sense, preventing infinite looping. It assumes that $FInf (N, \{C\})$ is finite whenever *N* is finite.

1. Repeat while $T \cup P \not = \emptyset$ and $\bot \notin Y \cup A$:
  1.1. Apply ComputeInfer or ChooseP to retrieve the next conclusion of an inference from *T* or the next formula from *P*, where *T* and *P* are organized as a single priority queue, to ensure fairness.
  1.2. Apply SimplifyFwd as long as the simplified formula $C'$ is $\succ$-smaller than the original formula *C*.
  1.3. If DeleteFwd is applicable, apply it.
  1.4. Otherwise:
    1.4.1. Apply DeleteBwd exhaustively.
    1.4.2. Apply SimplifyBwd as long as the simplified formula $C''$ is $\succ$-smaller than the original formula $C'$.
    1.4.3. Apply DeleteOrphans.
    1.4.4. Apply ScheduleInfer.

The instantiation of  relies on three labels  corresponding to the sets *P*, *Y*, *A*, respectively.

#### Example 82

Higher-order unification can give rise to infinitely many incomparable unifiers. As a result, in $\lambda$-superposition [14], performing all inferences between two clauses can lead to infinitely many conclusions, which need to be enumerated fairly. The Zipperposition prover [14], which implements the calculus, performs this enumeration in an extended DISCOUNT loop.

Infinitary inference rules are also useful to reason about the theory of datatypes and codatatypes. Superposition with (co)datatypes [19] includes *n*-ary Acycl and Uniq rules, which had to be restricted and complemented with axioms so that they could be implemented in Vampire [27]. In Zipperposition, it would be possible to support the rules in full generality, eliminating the need for the axioms.

Abstractly, a Zipperposition loop prover $\mathsf {ZL}$ operates on states $T \mid P \mid Y \mid A$, where *T* is organized as a finite set of possibly infinite sequences $(\iota _i)_i$ of inferences and the other components are as in $\mathsf {DL}$ (Example 81). The ChooseP, DeleteFwd, SimplifyFwd, DeleteBwd, and SimplifyBwd rules are essentially as in $\mathsf {DL}$. The other rules follow:

ComputeInfer: $$T \uplus \{(\iota _i)_i\} \mid P \mid \emptyset \mid A \,\Longrightarrow _\mathsf {ZL}\, T \cup \{(\iota _i)_{i\ge 1}\} \mid P \cup \{C\} \mid \emptyset \mid A$$
  if $$\iota _0 \in Red _{\text {I}}^{\cap {\mathcal {G}}}(A \cup \{C\})$$
ScheduleInfer: $$T \mid P \mid \{C\} \mid A \,\Longrightarrow _\mathsf {ZL}\, T \cup T' \mid P \mid \emptyset \mid A \cup \{C\}$$
  if $T'$ is a finite set of sequences $(\iota _i^j)_i^{j}$ of inferences such that the set of all $\iota _i^j$ equals $FInf (A, \{C\})$
DeleteOrphan: $$T \uplus \{(\iota _i)_i\} \mid P \mid Y \mid A \,\Longrightarrow _\mathsf {ZL}\, T \mid P \mid Y \mid A$$
  if $\iota _i \notin FInf (A)$ for all *i*

ComputeInfer works on the first element of sequences. ScheduleInfer adds new sequences to *T*. Typically, these sequences store $FInf (A, \{C\})$, which may be countably infinite, in such a way that all inferences in one sequence have identical premises and can be removed together by DeleteOrphan. The same rule can also be used to remove empty sequences from *T*, since the side condition is then vacuously true, thereby providing a form of garbage collection.

A subtle difference with $\mathsf {DL}$ is that ComputeInfer puts the formula *C* in *P* instead of *Y*. This gives more flexibility for scheduling; for example, a prover can pick several formulas from the same sequence and then choose the most suitable one—not necessarily the first one—to move to the active set.

To produce fair derivations, a prover needs to choose the sequence in ComputeInfer fairly and to choose the formula in ChooseP fairly. In combination, this achieves a form of dovetailing. The prover could use a simple algorithm, such as round-robin, for ComputeInfer and employ more sophisticated heuristics for ChooseP.

The implementation in Zipperposition uses a slightly more complicated representation for *T*, with sequences of subsingletons of inferences. Thus, each sequence element is either a single inference $\iota$ or the empty set, which signifies that no new unifier was found up to a certain depth.

#### Remark 83

The above approach to orphan formula deletion works because formulas recognized as orphans, belonging to the *T* state component, cannot have been used to make other formulas or inferences redundant—only passive and active formulas are considered by the redundancy criterion. If formulas from the *P* or *A* set could be detected as orphans, we could lose refutational completeness. To see this, consider the abstract scenario in which a formula *C* that is crucial for a refutation is subsumed by *D*, which is in turn deleted for being an orphan formula. Then *C* is lost forever even if is not an orphan formula.

Nevertheless, the idea of detecting orphan formulas outside the scheduled inferences in *T* can be salvaged as follows: Annotate each formula *D* with its parentage, and whenever *D* is used to simplify other clauses or to make other inferences redundant, remember this fact. Only consider *D* an orphan formula if it has lost a parent and if it has never beeen used to delete other formulas or to make other inferences redundant.

### Integrating Saturation Calculi

The prover architectures described above can be instantiated with saturation calculi that use a redundancy criterion obtained as an intersection of lifted redundancy criteria. Some saturation calculi are defined in such a way that this requirement is trivially satisfied. For others, some reformulation of the redundancy criterion may be necessary.

#### Example 84

As explained in Examples 54 and 55, redundancy criteria for calculi with selection functions [5, 6] or constraints [29, 30] can be defined as intersections $Red ^{\cap {\mathcal {G}}}$ of lifted redundancy criteria.

#### Example 85

In Bachmair and Ganzinger’s associative–commutative (AC) superposition calculus [4], the redundancy of general clauses and inferences is defined using a grounding function ${\mathcal {G}}$ that maps every clause *C* to the set of its ground instances $C\theta$ and every inference $\iota$ to the set of its ground instances $\iota \theta$. (“Instance” means “syntactic instance” here, that is, not “instance modulo AC.”) In principle, one could now apply $({\mathcal {G}},\sqsupset )$-lifting, where we choose $\sqsupset$ as the instantiation ordering modulo AC. This would be pointless, though, since in the definition of $Red _{\text {F}}^{{\mathcal {G}},\sqsupset }$ the ordering $\sqsupset$ is used only if *D* is a common syntactic instance of *C* and $C'$. Note that, for example, $C \,=\, {\mathsf {f}}({\mathsf {c}} + ({\mathsf {c}} + z)) \approx {\mathsf {b}}$ is an AC-instance of $C' \,=\, {\mathsf {f}}((x + x) + y) \approx {\mathsf {b}}$, but since *C* and $C'$ have no common syntactic ground instances, this fact is never exploited in $Red _{\text {F}}^{{\mathcal {G}},\sqsupset }$. We can repair this by redefining ${\mathcal {G}}$ so that it maps every $\iota$ to the set of its syntactic ground instances $\iota \theta$, as before, but *C* to the set of all *D* that are AC-equal to some ground instance $C\theta$. This qualifies as a grounding function as well, and since Bachmair and Ganzinger’s definition of redundancy for ground clauses is invariant under AC, the new definition of redundancy for general clauses is equivalent to the old one.

#### Example 86

Waldmann [44] considers a superposition calculus modulo $\Psi$-torsion-free cancellative abelian monoids. Redundant clauses and inferences are defined in the standard way by lifting, except for the Abstraction inference rule: According to Waldmann’s definition, a ground instance of an Abstraction inference $\iota = (C_2,C_1,C_0)$ is an Abstraction inference $(C_2\theta ,C_1\theta ,C_0\theta )$ where $C_2\theta$ and $C_1\theta$ are ground. But the conclusion of an Abstraction inference is never ground, and this applies also to $C_0\theta$. When defining redundancy for such inferences, it is therefore necessary to further instantiate the abstraction variable *y* in $C_0\theta$ using a substitution $\rho$ that maps *y* to a sufficiently small ground term. To obtain a grounding function ${\mathcal {G}}$ as defined in Sect. 3.1, we need to redefine ${\mathcal {G}}(\iota )$ as the set of all inferences $(C_2\theta ,C_1\theta ,C_0\theta \rho )$, rather than the set of all $(C_2\theta ,C_1\theta ,C_0\theta )$.

#### Example 87

The definition of redundancy for Bachmair, Ganzinger, and Waldmann’s hierarchic superposition calculus [8] is mostly standard, using a grounding function that maps every clause *C* to a subset ${\mathcal {G}}(C)$ of the set of its ground instances and every hierarchic superposition inference $\iota$ to a set ${\mathcal {G}}(\iota )$ of ground standard superposition inferences. There is one exception, namely, Close inferences, which derive $\bot$ from a list of premises that is inconsistent w.r.t. some base (background) theory. For these inferences, ${\mathcal {G}}(\iota ) = undef$.

Baumgartner and Waldmann’s variant of hierarchic superposition [10] uses a slightly different definition of redundancy: A clause *C* is redundant if ${\mathcal {G}}(C) \subseteq Red _{\text {F}}({\mathcal {G}}(N) \cup Th ) \cup Th$; a non-Close inference $\iota$ is redundant if ${\mathcal {G}}(\iota ) \subseteq Red _{\text {I}}({\mathcal {G}}(N) \cup Th )$, where $Th$ is a fixed set of ground base clauses and $Red$ is the usual redundancy criterion for ground standard superposition. To convert this into the format required in Sect. 3.1, we can define $Red _{\text {F}}^{ Th }(M) := Red _{\text {F}}(M \cup Th ) \cup Th$, and $Red _{\text {I}}^{ Th }(M) := Red _{\text {I}}(M \cup Th )$. It is easy to check that $Red ^{ Th } := ( Red _{\text {I}}^{ Th }, Red _{\text {F}}^{ Th })$ is also a redundancy criterion and that the properties above are equivalent to ${\mathcal {G}}(C) \subseteq Red _{\text {F}}^{ Th }({\mathcal {G}}(N))$ and ${\mathcal {G}}(\iota ) \subseteq Red _{\text {I}}^{ Th }({\mathcal {G}}(N))$. For Close inferences, we have again ${\mathcal {G}}(\iota ) = undef$.

#### Example 88

For saturation calculi whose refutational completeness proof is based on some kind of lifting of ground instances, the requirement to use a redundancy criterion obtained as an intersection of lifted redundancy criteria is rather natural. The outlier is unfailing completion [2].

Unfailing completion predates the introduction of Bachmair–Ganzinger-style redundancy, but it can be incorporated into that framework. The formulas are the rewrite rules and equations. The only inferences are orientation and critical pair computation; the other inferences of the unfailing completion calculus (e.g., simplifications of equations or rules) must be considered as simplifications in our framework, rather than as inferences. With these definitions, formulas and inferences are redundant if for every rewrite proof using that rewrite rule, equation, or critical peak, there exists a smaller rewrite proof.^5^

The requirement that the redundancy criterion must be obtained by lifting (which is necessary to introduce the labeling) can then be trivially fulfilled by “self-lifting”—i.e., by defining ${\mathbf {G}}:= {\mathbf {F}}$ and  and by taking ${\mathcal {G}}$ as the function that maps every formula or inference to the set of its $\alpha$-renamings.

Note that this definition of redundancy differs from the usual definition of redundancy for superposition. For example, with a term ordering satisfying ${\mathsf {f}}({\mathsf {c}}) \succ {\mathsf {f}}({\mathsf {b}}) \succ {\mathsf {f}}({\mathsf {a}}) \succ {\mathsf {c}} \succ {\mathsf {b}} \succ {\mathsf {a}}$, the equations ${\mathsf {c}} \approx {\mathsf {b}}$ and ${\mathsf {c}} \approx {\mathsf {a}}$ make ${\mathsf {f}}({\mathsf {b}}) \approx {\mathsf {f}}({\mathsf {a}})$ redundant in the superposition calculus (since they are smaller in the induced clause ordering), but they do not make ${\mathsf {f}}({\mathsf {b}}) \approx {\mathsf {f}}({\mathsf {a}})$ redundant in unfailing completion (since the rewrite proof ${\mathsf {f}}({\mathsf {b}}) \leftrightarrow {\mathsf {f}}({\mathsf {c}}) \leftrightarrow {\mathsf {f}}({\mathsf {a}})$ using ${\mathsf {c}} \approx {\mathsf {b}}$ and ${\mathsf {c}} \approx {\mathsf {a}}$ is larger than the rewrite proof ${\mathsf {f}}({\mathsf {b}}) \leftrightarrow {\mathsf {f}}({\mathsf {a}})$ using ${\mathsf {f}}({\mathsf {b}}) \approx {\mathsf {f}}({\mathsf {a}})$).

## Isabelle Development

The framework described in the previous sections has been formalized in Isabelle/HOL [31, 32], including all the theorems and lemmas, the prover architectures  and , and the example prover $\mathsf {RP}{}$. The Isabelle theory files are available in the *Archive of Formal Proofs* [16, 40]. The development is also part of the IsaFoL (Isabelle Formalization of Logic) [17] effort, which aims at developing a reusable computer-checked library of results about automated reasoning.

The main theory files of the development are listed below:

- Calculus.thy collects basic definitions and lemmas about consequence relations, inference systems, and redundancy criteria, including the equivalence of static and dynamic refutational completeness.
- Calculus_Variations.thy contains alternative notions of inferences, redundancy, saturation, and completeness found in the literature.
- Intersection_Calculus.thy introduces calculi equipped with a family of redundancy criteria, whose intersection is taken.
- Lifting_to_Non_Ground_Calculi.thy gathers the results on nonground liftings of calculi without and with well-founded orderings $\sqsupset _D$.
- Labeled_Lifting_to_Non_Ground_Calculi.thy contains the labeled extensions of the previous liftings.
- Given_Clause_Architectures.thy and Given_Clause_Architectures_Revisited.thy include results about the given clause prover  and its extension  with delayed inferences. The invariant-based proofs presented in this paper are found in the latter theory file.
- FO_Ordered_Resolution_Prover_Revisited.thy re-proves the ordered resolution prover $\mathsf {RP}$ refutationally complete using our framework, providing a modular alternative to the Isabelle formalization by Schlichtkrull et al. [36, 38].

The development relies heavily on Isabelle’s locales [9]. These are contexts that fix variables and make assumptions about these. Definitions and lemmas occurring inside the locale may then refer to them. With locales, the definitions and lemmas look similar to or even simpler than how they are stated on paper, but the proofs often become more complicated: Layers of locales may hide definitions, and often these need to be manually unfolded in several steps before the desired lemma can be proved. A pathological example is Lemma 64, which obviously holds by construction from a human perspective but whose Isabelle proof required more than a hundred lines of code.

We chose to represent basic nonempty sets such as ${\mathbf {F}}$ and ${\mathbf {L}}$ by types. This lightened the development in two ways. First, it relieved us from having to thread through nonemptiness conditions. Second, objects are automatically typed appropriately based on the context, meaning that lemmas could be stated without explicit hypotheses that given objects are formulas, labels, or indices. On the other hand, for sets such as ${\mathbf {F}}_\bot$ and $FInf$ that are subsets of other sets, it was natural to use simply typed sets. Derivations, which describe the dynamic behavior of a calculus, are represented by the same lazy list codatatype [18] and auxiliary definitions that were used by Schlichtkrull et al.

The framework’s design and its mechanization were carried out largely in parallel. This resulted in more work on the mechanization side because changes had to be propagated, but it also helped detect missing conditions and shape the theory itself. For example, an earlier version of the framework considered only single lifted redundancy criteria instead of intersections of lifted redundancy criteria (Sect. 3.3); our first attempt at verifying $\mathsf {RP}$ in Isabelle using the framework made it clear that the theory was not quite general enough yet to support selection functions (Example 54).

## Conclusion

We presented a formal framework for saturation theorem proving inspired by Bachmair and Ganzinger’s *Handbook* chapter [6]. Users can conveniently derive a dynamic refutational completeness result for a concrete prover based on a statically refutationally complete calculus. The key was to strengthen the standard redundancy criterion so that all prover operations, including subsumption deletion, can be justified by inference or redundancy. The framework is mechanized in Isabelle/HOL and can be used to verify actual provers.

To employ the framework, users must provide a statically complete saturation calculus expressible as the lifting $( FInf , Red ^{\mathcal {G}})$ or $( FInf , Red ^{\cap {\mathcal {G}}})$ of a ground calculus $( GInf , Red )$, where $Red$ qualifies as a redundancy criterion and ${\mathcal {G}}$ qualifies as a grounding function or grounding function family. The framework can be used to derive two main results:

1. After defining a well-founded ordering $\sqsupset$ or a family of well-founded orderings that capture instantiation, invoke Theorem 53 to show $( FInf , Red ^{\cap {\mathcal {G}},\sqsupset })$ dynamically complete.
2. Based on the previous step, invoke Theorems 70 or 80 to derive the dynamic completeness of a prover architecture building on the given clause procedure, such as the Otter, iProver, DISCOUNT, or Zipperposition loop (Examples 71, 74, 81, or 82).

The framework can also help establish the static completeness of the nonground calculus. For many calculi (with the notable exceptions of constraint and hierarchic superposition), Theorems 32 or 50 can be used to lift the static completeness of $( GInf , Red )$ to $( FInf , Red ^{\mathcal {G}})$ or $( FInf , Red ^{\cap {\mathcal {G}}})$.

The main missing piece of the framework is a generic treatment of clause splitting. Until recently, the only formal treatment of splitting, by Fietzke and Weidenbach [22], hard-codes both the underlying calculus and the splitting strategy. Voronkov’s AVATAR architecture [42] is more flexible and yields truly impressive empirical results, but he and his collaborators left the question of AVATAR’s refutational completeness open. Ebner et al. [21] recently provided an answer by introducing and instantiating a generic splitting framework based on our saturation framework.

## Footnotes

1 Note that Bachmair and Ganzinger’s *J. Log. Comput.* article [5] uses a terminology that differs from most later publications in this area: Their “composite” corresponds to “redundant,” and their “complete” corresponds to “saturated.”

2 The limit need not be saturated, though. In Example 17, the one-element sequence $(N_i)_{i=0}^0$ with $N_0 = N$ is reducedly fair w.r.t. $Red$, and its limit $N_\infty = N$ is reducedly saturated w.r.t. $Red$, but not saturated w.r.t. $Red$.

3 We could also use a *Q*-indexed family of sets $({\mathbf {G}}^q)_{q \in Q}$ instead of a single set ${\mathbf {G}}$, and a $(Q,{\mathbf {G}}^q)$-indexed family of well-founded strict partial orderings on ${\mathbf {F}}$ instead of a ${\mathbf {G}}$-indexed family, but we are not aware of applications where this is necessary.

4 We keep the traditional term “given clause procedure” even though our framework is not restricted to clauses.

5 Proofs are compared using a proof ordering—that is, the multiset extension of an ordering that compares individual proof steps $s \rightarrow _R t$, $s \leftarrow _R t$, or $s \leftrightarrow _E t$.
